# The Gas-Phase Formation Mechanism of Dibenzofuran (DBF), Dibenzothiophene (DBT), and Carbazole (CA) from Benzofuran (BF), Benzothiophene (BT), and Indole (IN) with Cyclopentadienyl Radical

**DOI:** 10.3390/ijms20215420

**Published:** 2019-10-31

**Authors:** Xuan Li, Yixiang Gao, Chenpeng Zuo, Siyuan Zheng, Fei Xu, Yanhui Sun, Qingzhu Zhang

**Affiliations:** 1 Environment Research Institute, Shandong University, Qingdao 266237, China; lx18265450270@126.com (X.L.); yixianggao@163.com (Y.G.); zuochenpeng@126.com (C.Z.); zhengsiyuan1991@126.com (S.Z.); zqz@sdu.edu.cn (Q.Z.); 2 Shenzhen Research Institute, Shandong University, Shenzhen 518057, China; 3 College of Environment and Safety Engineering, Qingdao University of Science & Technology, Qingdao 266042, China; sunyh0532@126.com

**Keywords:** nso-hets, formation mechanism, cyclopentadienyl, rate constant, density functional method

## Abstract

Benzofuran (BF), benzothiophene (BT), indole (IN), dibenzofuran (DBF), dibenzothiophene (DBT), and carbazole (CA) are typical heterocyclic aromatic compounds (NSO-HETs), which can coexist with polycyclic aromatic hydrocarbons (PAHs) in combustion and pyrolysis conditions. In this work, quantum chemical calculations were carried out to investigate the formation of DBF, DBT, and CA from the reactions of BF, BT, and IN with a cyclopentadienyl radical (CPDyl) by using the hybrid density functional theory (DFT) at the MPWB1K/6-311+G(3df,2p)//MPWB1K/6-31+G(d,p) level. The rate constants of crucial elementary steps were deduced over 600−1200 K, using canonical variational transition state theory with a small-curvature tunneling contribution (CVT/SCT). This paper showed that the production of DBF, DBT, and CA from the reactions of BF, BT, and IN with CPDyl involved six elementary steps: the addition reaction, ring closure, the first H shift, C–C cleavage, the second H shift, and elimination of CH_3_ or H. The cleavage of the C–C bond was regarded as the rate-determining step for each pathway due to the extremely high barrier. The 1-methyl substituted products were more easily formed than the 4-methyl substituted products. The main products were DBF and 1-methyl-DBF, DBT and 1-methyl-DBT, and CA and 1-methyl-CA for reactions of BF, BT, and IN with CPDyl, respectively. The ranking of DBF, DBT, and CA formation potential was as follows: DBT and methyl-DBT formation > DBF and methyl-DBF formation > CA, and methyl-CA formation. Comparison with the reaction of naphthalene with CPDyl indicated that the reactions of CPDyl attacking a benzene ring and a furan/thiophene/pyrrole ring could be inferred to be comparable under high temperature conditions.

## 1. Introduction

Polycyclic aromatic compounds (PACs), including polycyclic aromatic hydrocarbons (PAHs) and heterocyclic aromatic compounds (NSO-HETs), have become a considerable threat to the environment and human health because of their acute toxicity, mutagenicity, photoinduced toxicity, and carcinogenic potential [1,2]. The emissions of PAHs are often associated with releases of NSO-HETs, which have been reported to comprise up to 10% and 40% of the total PAH emissions in tar-oil or coal tar and its water-soluble fraction, respectively [3,4,5,6].

Benzofuran (BF), benzothiophene (BT), indole (IN), dibenzofuran (DBF), dibenzothiophene (DBT), and carbazole (CA) are typical NSO-HETs, which are composed of a benzene ring and a furan/thiophene/pyrrole ring. Furan/thiophene/pyrrole rings are five-membered ring in which the heteroatoms have at least one pair of non-binding valence shell electrons. BF, BT, and IN (benzo-NSO-HETs) include one benzene ring and one heterocyclic five-membered ring; DBF, DBT, and CA (dibenzo-NSO-HETs) consist of two benzene rings fused together on either side of a furan/thiophene/pyrrole ring, respectively. They have been found to be toxic and mutagenic [7]. DBF has been used as an insecticide, a component in heat-transfer oils, and a carrier for dyeing and printing textiles [8,9]. DBT is an important intermediate in the production of cosmetics and pharmaceuticals, and a model compound in the hydrodesulfurization reaction for diesel oil. CA has been widely used in insecticides, explosives, dyes, reagents, lubricants, and detergents [10,11,12], and in the pharmaceutical industry to treat encephalopathy, cardiopathy, hepatopathy, and arteriosclerosis [10,13]. Dibenzo-NSO-HETs are widely found in creosote, coal tar, and crude oils, as well as in some high temperature processes of waste incineration, tobacco smoke, aluminum manufacturing, forest fire, rubber, petroleum, fossil fuel, and coal and wood combustion [7,14,15,16,17,18], and always coexist with other aromatic compounds, such as PAHs and benzo-NSO-HETs [15,19,20,21,22,23,24,25]. Wikstrom reported that a high concentration of DBF was measured in artificial municipal solid waste fuel, and that the concentration of DBF (19,800 ng/nm^3^) was 100–1000 times and 10–100 times higher than that of PCDFs before and after the convector section, respectively [26]. DBF (190−210 ng/g), DBT (190 ng/g), and CA (1.5–1.6 ng/g) were detected by Ishikawa in nine types of industrial wastes and their pyrolytic decomposition products [19]. Gu reported that the concentration of DBT was 50−100 times higher than that of DBT in coal pyrolysis, where the highest concentration was found at 500−600 °C [27]. Methyl-DBF, methyl-DBT, and methyl-CA can be widely detected along with DBF, DBT, and CA in sediment, ground water, oil fuel, and other thermal process [1,28,29,30,31,32,33]. For example, DBT and 18 alkyl homologs were detected in coastal sediments in Qatar, and the concentration of alkylated DBT was 10−50 times than that of DBT [29]. Three isomers of methyl-DBT (1-methyl-DBT, 2 or 3-metyl-DBT, and 4-methyl-DBT), were identified by Rebbert in crude oil, in which 4-methyl-DBT was the obtained with the highest precision at a concentration of 33.9 µg/g [28].

Recently, several studies have revealed a considerably stronger relationship of dibenzo-NSO-HET and benzo-NSO-HET mass concentrations or isomer distributions in thermal processes. For example, Dartiguelongue found that DBT thermal cracking led to the formation of BT, phenyl-DBT, and other sulfur-containing molecules under isothermal pyrolysis conditions (375−500 °C) [27]. Sobkowiak reported a linear correlation between fuel phenol concentration with IN and CA in fuel-improved thermal oxidation under 1000 °C [21]. The formation enthalpies of 4-methylbenzofuran + naphthalene → dibenzofuran + toluene and 4-methylbenzofuran + anthracene → dibenzofuran + 1-methylnaphthalene were calculated at G3(MP2)//B3LYP to be −22.5 and −21.1 Kj/mol by Freitas at the fusion and combustion temperatures, respectively [22]. The cyclopentadienyl radical (C_5_H_5_, CPDyl) is among the most abundant radicals formed during the combustion and pyrolysis of many hydrocarbons [34,35,36,37,38]. Winkler studied the gas-phase pyrolysis of heterocyclic compounds in the flow pyrolysis and annulation reactions of some sulfur heterocycles, and found that DBF was produced from the reaction of BF with C_4_H_2_ above 850 °C [22]. Xing focused on the release behavior of thiophenic sulfur compounds during coal pyrolysis, and obtained levels of benzothiophene and dibenzothiophene during pyrolysis at temperatures ranging from 200 °C to 1300 °C, with the peak values reached at 800 °C. The amount of benzothiophene and dibenzothiophene released was much higher at a rapid pyrolysis rate than at a medium heating rate [22].

The CPDyl radical can be produced in high-temperature conditions by pyrolysis or unimolecular decomposition reactions of cyclopentadiene (C_5_H_6_) → C_5_H_5_ + H, phenoxy radical (C_6_H_5_O) → C_5_H_5_ + CO and acetylene (C_2_H_2_) + propargyl radical (C_3_H_3_) → C_5_H_5_. The experimental rate constants of C_5_H_6_ → C_5_H_5_ + H and C_6_H_5_O → C_5_H_5_ + CO are 2.3 × 10^1^ s^−1^ and 2.5 × 10^3^ s^−1^, respectively [19,34,35,36,39,40,41,42,43,44]. Among many potential precursors for aromatic growth, CPDyl is considered to be important in the formation and growth of PAHs, because it is neutral, ambidentally reactive at different sites, and has the ability of self-recombination [19,34,35,36,39,40,41,42,43,44]. Our recent studies proposed a detailed PAH growth mechanism from the reaction of naphthalene with CPDyl, followed by conversion of five-membered rings to six-membered rings, and found that the formation potential of bend products was larger than that of linear products [45]. Considering the structure and property similarities of dibenzo-NSO-HETs and benzo-NSO-HETs, and the coexistence of dibenzo-NSO-HETs and benzo-NSO-HETs with PAHs, the formation pathways of dibenzo-NSO-HETs from benzo-NSO-HETs with CPDyl were inferred to be similar to the growth mechanisms of PAH with CPDyl, where the five-membered ring of CPDyl can convert to a benzene ring followed by the addition reaction to benzo-NSO-HETs. As far as we know, there is no information available on dibenzo-NSO-HET formation mechanisms from benzo-NSO-HETs with the CPDyl radical.

Due to the high toxicity of dibenzo-NSO-HETs and the lack of efficient detection methods for intermediate radicals, the detailed formation mechanism of dibenzo-NSO-HETs from benzo-NSO-HETs with CPDyl has not been completely elucidated. Quantum chemical calculations can be widely used to predict favorable reaction pathways and elucidate the yield of the products, and theoretical modeling allows direct contact with the highly toxic compounds to be avoided. In this study, we present an overall density functional theory (DFT) study of dibenzofuran (DBF), dibenzothiophene (DBT), and carbazole (CA) formation mechanisms from the reactions of benzofuran (BF), benzothiophene (BT), and indole (IN) with CPDyl, respectively. All possible formation pathways involved in dibenzo-NSO-HET formation from benzo-NSO-HETs with the CPDyl radical were studied. Some energetically preferred routes were proposed to parallel the formation possibility of different dibenzo-NSO-HET products and explain experimental observations. Secondly, rate constants for the elementary reactions were evaluated over a wide temperature range of 600−1200 K. The absence of the kinetic parameters prevent further improvement and optimization of the NSO-HETs formation models. The third aim was to compare the effect of the O, S, and N atom substitution pattern of benzo-NSO-HETs on the isomer patterns and formation potential of dibenzo-NSO-HETs.

## 2. Results

**Figure 1:**
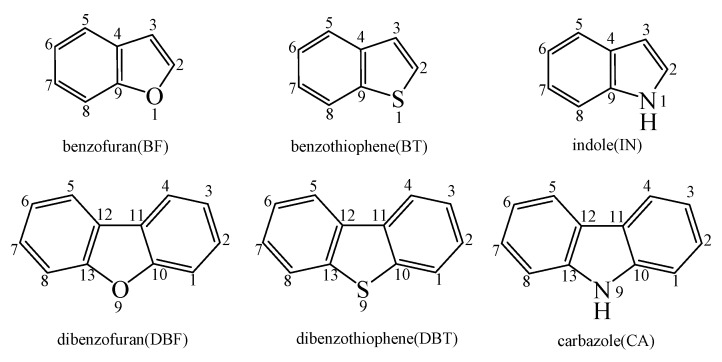
The structures of benzofuran (BF), benzothiophene (BT), indole (IN), dibenzofuran (DBF), dibenzothiophene (DBT), and carbazole (CA) with labeled C atoms.

For convenience of interpretation, the C atoms of benzofuran (BF), benzothiophene (BT), indole (IN), dibenzofuran (DBF), dibenzothiophene (DBT), and carbazole (CA) were labeled, as presented in Figure 1.

It is crucial to clarify the reliability and accuracy of the theoretical calculations. The geometric parameters and the vibrational frequencies of BF, BT, IN, DBF, DBT, and CA were calculated at the MPWB95/6-31+G(d,p) level and compared with the experimental values in Tables S3–S5 of Supporting Information. The results at the MPWB95/6-31+G(d,p) level were in accordance with the experimental data, and the average relative error remained within 3.3% for the geometrical parameters [46,47,48,49,50], and less than 5.7% for the vibrational frequencies of BF, BT, IN, DBF, DBT, and CA [51,52,53,54]. To verify the reliability of the energies, we calculated the reaction heats for benzofuran (C_8_H_6_O) + cyclopentadiene (C_5_H_6_) → dibenzofuran (C_12_H_8_O) + CH_4_, benzothiophene (C_8_H_6_S) + cyclopentadiene (C_5_H_6_) → dibenzothiophene (C_12_H_8_S) + CH_4_, and indole (C_8_H_7_N) + cyclopentadiene (C_5_H_6_) → carbazole (C_12_H_9_N) + CH_4_ at the MPWB1K/6-311+G(3df,2p)//MPWB1K/6-31+G(d,p) level. The calculated values of −41.5 kcal/mol, −39.7 kcal/mol, and −40.1 kcal/mol at 298 K showed good consistency with the corresponding experimental values of −43.0 kcal/mol, −39.8 kcal/mol, and −38.3 kcal/mol obtained from the measured standard formation heats(∆*H*_f,0_) of BF, BT, IN, DBF, DBT, CA, and CPDyl [55,56,57,58,59,60,61,62]. From these results, we inferred that accuracy can be expected for the species in this study.

### 2.1. Reaction of Benzofuran, Benzothiophene, and Indole with Cyclopentadienyl

**Figure 2:**
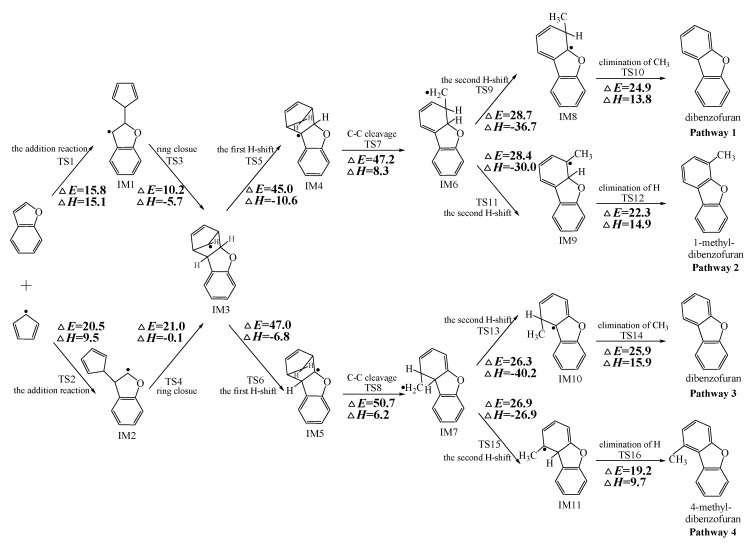
Dibenzofuran (DBF) formation routes presented with the activation barriers Δ*E* (in kcal/mol) and reaction heats Δ*H* (in kcal/mol) in the reaction of benzofuran (BF) with cyclopentadienyl radical (CPDyl). Δ*H* has been calculated at 0 K.

**Figure 3:**
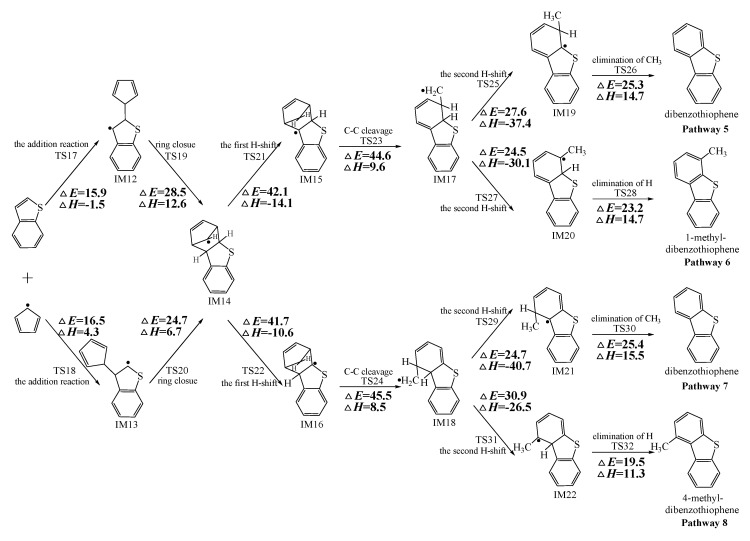
Dibenzothiophene (DBT) formation routes embedded with the activation barriers Δ*E* (in kcal/mol) and reaction heats Δ*H* (in kcal/mol) in the reaction of benzothiophene (BT) with cyclopentadienyl radical (CPDyl). Δ*H* has been calculated at 0 K.

**Figure 4:**
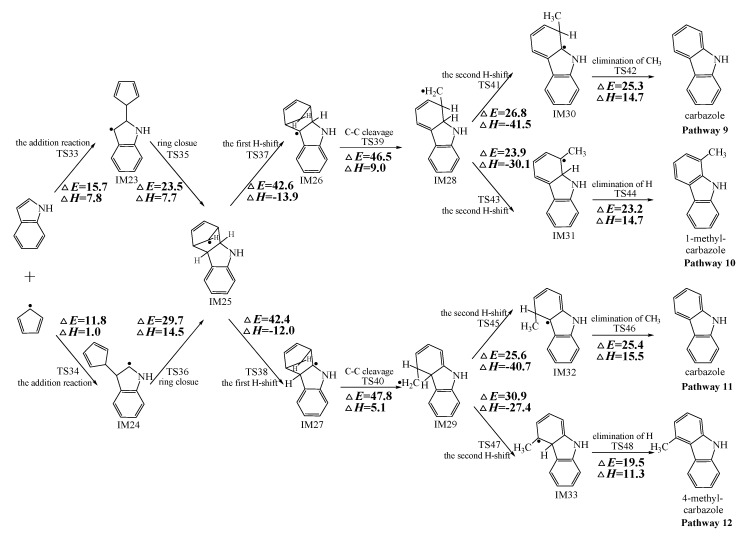
Carbazole (CA) formation routes embedded with the activation barriers Δ*E* (in kcal/mol) and reaction heats Δ*H* (in kcal/mol) in the reaction of indole (IN) with cyclopentadienyl radical (CPDyl). Δ*H* has been calculated at 0 K.

Figure 2, Figure 3 and Figure 4 schematically illustrate the proposed reaction pathways for dibenzofuran (DBF), dibenzothiophene (DBT), and carbazole (CA) formation mechanisms from the reactions of benzofuran (BF), benzothiophene (BT), and indole (IN) with the cyclopentadienyl radical (CPDyl), respectively. The activation barriers Δ*E* (in kcal/mol) and reaction heats Δ*H* (in kcal/mol) were calculated at the MPWB1K/6-311+G(3df,2p)//MPWB1K/6-31+G(d,p) level. The activation barrier Δ*E* is the relative energy of the transition state with respect to the total energy of the separated reactants. The reaction heats Δ*H* is the relative energy of total energy of the products with respect to the total energy of reactants. Positive values of Δ*H* indicate endothermic reactions, and negative values indicate exothermic reactions. The relative activation barriers Δ*E_r_* (in kcal/mol) and relative reaction heats Δ*H_r_* (in kcal/mol) for all the elementary reactions in Figure 2, Figure 3 and Figure 4 at the MPWB1K/6-311+G(3df,2p)//MPWB1K/6-31+G(d,p) level are shown in Table S2 of the Supporting Information. The relative activation barrier Δ*E_r_* is the relative energy of the transition state with respect to the total energy of the BF/BT/IN and CPDyl. The relative reaction heat Δ*H_r_* is the relative energy of the total energy of the products with respect to the total energy of the BF/BT/IN and CPDyl. Four possible pathways (denoted as pathways 1−4 in Figure 2, pathways 5−8 in Figure 3, and pathways 9−12 in Figure 4) are postulated in each figure, resulting in three oxygen heterocycle dibenzo-NSO-HET congeners (DBF, 1-methyl-DBF, and 4-methyl-DBF) in Figure 2, three sulfur heterocycle dibenzo-NSO-HET congeners (DBT, 1-methyl-DBT, and 4-methyl-DBT) in Figure 3, and three nitrogen heterocycle dibenzo-NSO-HET congeners (CA, 1-methyl-CA, and 4-methyl-CA) in Figure 4.

The CPDyl radical has an unpaired electron, while the benzo-NSO-HET molecule has C=C conjugated π bonds. Thus, the reaction of benzo-NSO-HETs with the CPDyl radical can proceed through an addition reaction. The C atoms in benzo-NSO-HETs can be divided into three groups: C2 and C3 belong to the five-membered ring; C4 and C9 are inside the “bend” of the benzo-NSO-HET molecules; C5, C6, C7, and C8 belong to the six-membered benzene ring. Among them, CPDyl additions to C4 and C9 are sterically hindered, and additions to C5, C6, C7, and C8 could not form the dioxin-like dibenzo-NSO-HETs. Our previous work studied the formation and growth mechanism of PAHs from the reactions of naphthalene with CPDyl, and found that the potential barriers of the CPDyl addition to aromatic benzene ring are 16.3 and 18.4 kcal/mol: close to the 11.8–16.5 kcal/mol of the CPDyl addition to the five-membered furan/thiophene/pyrrole rings, except the energetically unfavored 20.5 kcal/mol of BF. This indicates that CPDyl addition to the six-membered ring (C5–C8) and five-membered ring (C2–C3) is energetically competitive. However, only CPDyl additions to C2 and C3 of benzo-NSO-HETs can form dibenzo-NSO-HET products. Thus, in this study, CPDyl additions to C2 and C3 of the furan ring of BF (resulting in IM1 and IM2), CPDyl additions to C2 and C3 of the thiophene ring of BT (resulting in IM12 and IM13), and CPDyl additions to C2 and C3 of the pyrrole ring of IN (resulting in IM23 and IM24) are discussed in detail in Figure 2, Figure 3, and Figure 4, respectively. In Figure 1, calculations show that the C2 and C3 additions via TS1 and TS2 have high activation barriers of 15.8 kcal/mol and 20.5 kcal/mol, respectively, indicating that CPDyl addition to C2 of BF is energetically preferred compared to addition to C3 of BF. Similarly, CPDyl addition to C2 of BT can occur via 15.9 kcal/mol, which is lower than the addition to C3 of BT. However, CPDyl addition to C2 of IN has a much higher activation barrier (15.7 kcal/mol) than CPDyl addition to C3 (11.8 kcal/mol) of IN, which means that CPDyl addition to C3 can take place much more readily than to C2 in CA formation from IN with CPDyl. After the addition reactions, IM1/IM2, IM12/IM13, and IM23 /IM24 undergo the ring close step to form the same intermediates, IM3, IM14, and IM25, respectively, which means that the first two steps of the different pathways in each figure are identical. The structures with the geometrical parameters of transition states and intermediates involved in this study are shown Figures S1–S3 of the Supporting Information, and the cartesian coordinates for transition states and intermediates are displayed in Tables S7 and S8 of the Supporting Information.

**Figure 5:**
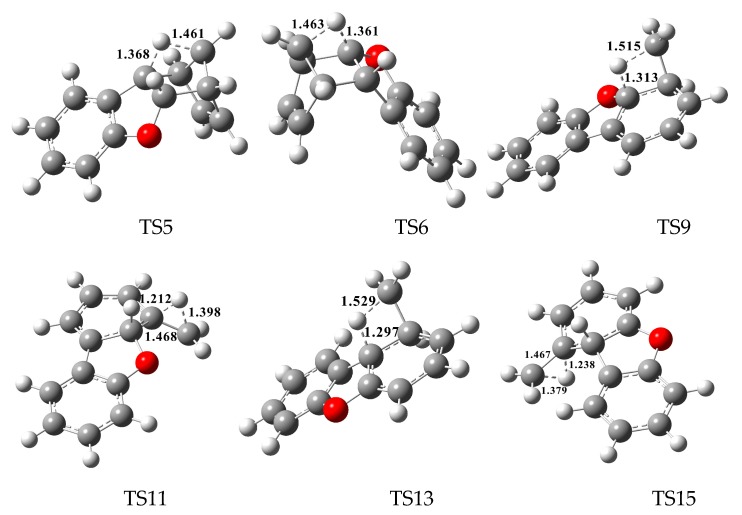
The structures and the geometrical parameters for the first/second H-shift transition states in the dibenzofuran (DBF) formation routes from the reaction of benzofuran (BF) with cyclopentadienyl radical (CPDyl). Distances are in angstroms.

In Figure 2, Figure 3 and Figure 4, each pathway is comprised of six elementary steps: (1) the addition reaction; (2) ring closure; (3) the first H shift; (4) C–C cleavage; (5) the second H shift, and (6) unimolecular elimination of CH_3_ or H. All of the H-shift steps are strongly exothermic, while the other steps are endothermic. The C–C cleavage steps and the first H-shift steps need to overcome high activation barriers exceeding 40 kcal/mol. At high-temperature conditions in combustion and thermal processes, these activation barriers are easily overcome. For each pathway, the C–C cleavages are the rate-determining steps owing to their high activation barriers (47.2 kcal/mol and 50.7 kcal/mol in Figure 2, 44.6 kcal/mol and 45.5 kcal/mol in Figure 3, and 46.5 kcal/mol and 47.8 kcal/mol in Figure 4). The structures, along with the geometrical parameters for the first/second H-shift transition states in the DBF formation routes from BF with CPDyl are shown in Figure 5. In Figure 2 and Figure 5, pathways 1−2 can occur from via the same first H-shift step, where the H atom bonded to the C-*ortho* bend C (C11) transfers to the bridge C of DF (the first C11–H shift); pathways 3−4 can occur via the same first H-shift step, where the H atom bonded to the O-*ortho* bend C (C11) transfers to the bridge C of DF (the first C10–H shift). Similarly, pathways 5−6 in Figure 3 and pathways 9–10 in Figure 4 arise from the first C11–H shift, whereas pathways 7−8 in Figure 3 and pathways 11−12 in Figure 4 arise from the first C10–H shift. In Figure 2, pathways 1–2 can occur via the same intermediate IM6, and the distinctions of pathways 1–2 lie in the final two elementary steps, the second H shift and the elimination of CH_3_/H. Similarly, pathways 3–4 have the same intermediate IM7, with the last two steps being difference. In pathways 1 and 3, the second H-shift steps are identical, where the H atom bonded to the same carbon atom (with –CH_2_) transfers to –CH_2_ (the second *same* C–H shift), resulting in the elimination of CH_3_ in the last step. Thus, the main products of pathways 1 and 3 are DBF without methyl substitution. However, in pathways 2 and 4, the second H-shift steps were similar, where the H atom bonded to the *ortho* carbon atom (inside the bend) transfers to –CH_2_ (the second *ortho* C–H shift), resulting in the elimination of H and retaining the methyl in the last step. Thus, the main products of pathway 2 and 4 are methyl-DBF, i.e., 1-methyl-DBF and 4-methyl-DBF. From Figure 5, the second H shift in pathways 1 and 3 can occur via a four-member ring transition state, while the second H shift in pathways 2 and 4 can occur via a three-member ring transition state.

### 2.2. Rate Constant Calculations

It is difficult to measure the rate constants of the elementary reactions by experimental methods in the formation of dibenzo-NSO-HETs, especially the reactions involving the radicals and intermediates due to the short lifetime and lack of efficient detection schemes for these activated species. Direct dynamics calculations, that is, the calculation of rate constants or other dynamic information directly from electronic structure calculations can be used as a substitution. The kinetic parameters of dibenzo-NSO-HETs formation routes from benzo-NSO-HETs with CPDyl can be used in the NSO-HET compound formation kinetic model to predict the potential emissions and harm to the environment. In this section, the rate constants of the elementary reactions for the formation of dibenzo-NSO-HETs from benzo-NSO-HETs with CPDyl were calculated using canonical variational transition state theory (CVT) with small-curvature tunneling (SCT) contribution over 600–1200 K by means of the POLYRATE 9.7 program [63,64,65,66,67]. The calculated rate constants are shown in Table S6 of the Supporting Information for every 100 K in 600–1200 K.

Due to the absence of available experimental values, it was difficult to make a direct comparison of the calculated CVT/SCT rate constants with the experimental values for all the elementary reactions. In order to clarify the reliability of the kinetics calculation in this study, we compared the CVT/SCT rate constants with the rate constant values available in the literature for the reaction of C_6_H_5_O → C_5_H_5_ + CO. The rate constants measured in previous experiments were in excellent agreement with our CVT/SCT values. For example, at 1200 K, the CVT/SCT rate constant measured by Lin was 2.5 × 10^3^ s^−1^ [68], which agreed well with the value of 2.6 × 10^3^ s^−1^ found in this study. The rate constants of the rate-determining steps in Figure 2, Figure 3 and Figure 4 were calculated with the help of transition state theory (TST) by using Gibbs thermal corrections at high temperatures in combination with the Eyring equation [45,69]. In general, the TST rate constants were slightly larger than the CVT/SCT values. For example, at 1000 K, the TST rate constants were 2.2 × 10^3^ s^−1^, 8.0 × 10^3^ s^−1^, and 2.8 × 10^3^ s^−1^ of the rate-determining steps for DBF, DBT, and CA formation, respectively, while the CVT/SCT values were 8.1 × 10^2^ s^−1^, 6.6 × 10^3^ s^−1^, and 1.2 × 10^3^ s^−1^ for DBF, DBT, and CA formation, respectively. The CVT/SCT method was also successfully used in polychlorinated dibenzofuran (PCDFs) and polychlorinated dibenzo-p-dioxin (PCDDs) formation from CPs, and polychlorinated dibenzothiophene (PCDTs) and polychlorinated thianthrene (PCTAs) formation from CTPs in our previous studies [45,69]. We hope our CVT/SCT calculations can provide the same excellent estimation accuracy for the crucial elementary reactions involved in the formation of dibenzo-NSO-HETs.

To improve the applicability, the rate constants were fitted, and Arrhenius formulas are given in Table 1 for the DBF formation routes from the reaction of BF with CPDyl, Table 2 for the DBT formation routes from the reaction of BT with CPDyl and Table 3 for the CA formation routes from the reaction of IN with CPDyl. The pre-exponential factor, the activation energy, and the rate constants can be obtained from these Arrhenius formulas.

## 3. Discussion

### 3.1. Reactions of Benzofuran, Benzothiophene, and Indole with Cyclopentadienyl

As presented in Figure 2, pathways 1−2 from the first C11–H shift step shared an identical rate-determining reaction (C–C cleavage), and pathways 3−4 from the first C10–H shift step also shared the same rate-determining reactions (C–C cleavage). The activation barrier of the rate-determining step of pathways 1−2 involved in the reaction of BF with CPDyl radical processed amounted to 47.2 kcal/mol, which was generally lower than that of the rate-determining step (50.7 kcal/mol) in pathways 3−4. Therefore, pathways 1−2, forming DBF and 1-methyl-DBF, were enthalpically preferable over pathways 3−4, forming DBF and 4-methyl-DBF, in Figure 2. Thus, 1-methyl-DBF was much easier to generate than 4-methyl-DBF. Similar conclusions were obtained in the reactions of BT with CPDyl in Figure 3 and of IN with CPDyl in Figure 4. The C–C cleavage in pathways 5−6, resulting in the formation of DBT and 1-methyl-DBT, had a relatively lower activation barrier (44.6 kcal/mol) than that in pathways 7−8, resulting in the formation of DBT and 4-methyl-DBT, which indicated that pathways 5−6 are more labile than pathways 7−8 in the reaction of DBT with CPDyl. Analogously, pathways 9−10, forming CA and 1-methyl-CA, were overwhelmingly superior to pathways 11 and 12, forming CA and 4-methyl-CA, in the reaction of In with CPDyl in Figure 4, and the activation barriers of the C–C cleavage step were 46.5 and 47.8 kcal/mol in pathways 9−10 and pathways 11−12, respectively. Therefore, 1-methyl-DBT and 1-methyl-CA were more easily formed than 4-methyl-DBT and 4-methyl-CA, respectively.

Comparing pathway 1 and pathway 2, the activation barrier of the second *ortho* C–H shift in pathway 2 (28.4 kcal/mol) was lower than that of the second *same* C–H shift in pathway 1 (28.7 kcal/mol). In addition, the elimination of H step in pathway 2 required a lower activation barrier of (22.3 kcal/mol) to be overcome than that of elimination of CH_3_ in pathway 1(24.9 kcal/mol). Thus, pathway 2, resulting in 1-methyl-DBF formation, was overwhelmingly superior to pathway, 1 resulting in the DBF formation. For pathways 3–4, the second *same* C–H shift step involved in the pathway 3 (activation barrier 26.3 kcal/mol, reaction heat −40.2 kcal/mol) had a relatively lower barrier and was much more exothermic compared to the second *ortho* C–H shift step involved in pathway 4 (activation barrier 26.9 kcal/mol, reaction heat −26.9 kcal/mol). However, the unimolecular elimination of CH_3_ involved in pathway 3 (activation barrier 25.9 kcal/mol, reaction heat 15.9 kcal/mol) had a relatively higher barrier and was much more endoergic than the unimolecular elimination of H involved in pathway 4 (activation barrier 19.2 kcal/mol, reaction heat 9.7 kcal/mol). Thus, pathway 3 and pathway 4 were competitive, which means that both DBF and 4-methyl-DBF can be formed competitively. Above all, although 1-methyl-DBF formation was preferred over DBF formation from the view of energetic values, 1-methyl-DBF formation had one less formation route than DBF formation. Thus, 1-methyl-DBF and DBF were inferred to be produced competitively, and to be more liable to form than 4-methyl-DBF. Further direct experimental observation will be needed to verify the yields of DBF, 1-methyl-DBF, and 4-methyl-DBF. Analogously, pathway 6 was favored over pathway 5 in Figure 3; pathway 7 and pathway 8 were competitive in Figure 3; pathway 10 was favored over pathway 9 in Figure 4; and pathway 11 and pathway 12 were competitive in Figure 4. Therefore, the main products of DBT formation from the reaction of BT with CPDyl are DBT and 1-methyl-DBT, and the main products of CA formation from the reaction of IN with CPDyl are carbazole and 1-methyl-CA.

Comparison of DBF and methyl-DBF formation from BF with CPDyl, DBT and methyl-DBT formation from BT with CPDyl, and CA and methyl-CA formation from IN with CPDyl, as denoted in Figure 2, Figure 3 and Figure 4, clearly showed that the effect of the O, S, and N atom heterocycle of the benzo-NSO-HETs had a significant influence on dibenzo-NSO-HET formation. For the pathways from the first C11–H shift in Figure 2, Figure 3 and Figure 4, the activation barriers of the rate-determining step of O heterocycle pathways, S heterocycle pathways, and N heterocycle pathways were 47.2 kcal/mol, 44.6 kcal/mol, and 46.5 kcal/mol, respectively. In addition, for the pathways from the first C10–H shift in Figure 2, Figure 3 and Figure 4, the activation barriers of the rate-determining steps of O heterocycle pathways, S heterocycle pathways, and N heterocycle pathways were 50.7 kcal/mol, 45.5 kcal/mol, and 47.8 kcal/mol, respectively. Thus, the ranking of the dibenzo-NSO-HET formation potential is as follows: sulfureted heterocycle compounds > nitrided heterocycle compounds > oxygenated heterocycle compounds, i.e., DBT and methyl-DBT formation > DBF and methyl-DBF formation > CA and methyl-CA formation. It is also interesting to compare the reaction pathways denoted in Figure 2, Figure 3 and Figure 4 with our previous research on naphthalene with CPDyl [45]. The activation barriers of the rate-determining steps for phenanthrene and methyl-phenanthrene formation from the reaction of naphthalene with CPDyl were 42.0–43.2 kcal/mol, i.e., lower than those of dibenzo-NSO-HET formation from benzo-NSO-HETs with CPDyl (44.6–50.7 kcal/mol). However, the activation barriers of the rate-determining steps for anthracene and methyl-anthracene formation (52.9 kcal/mol) were higher than those of dibenzo-NSO-HET formation from benzo-NSO-HETs with CPDyl. This may imply that the reactions of CPDyl attacking benzene rings are enthalpically competitive with reactions of CPDyl added onto furan/thiophene/pyrrole rings under pyrolysis or combustion conditions.

### 3.2. Rate Constant Calculations

To confirm the possible routes of dibenzo-NSO-HET formation from benzo-NSO-HETs with CPDyl, it is important to compare the CVT/SCT rate constants of the rate-determining step in each pathway. At a given temperature, the calculated CVT/SCT rate constants for the rate-determining steps in pathways 1–2, pathways 5–6, and pathways 9–10 were larger than those of pathways 3–4, pathways 7–8, and pathways 11–12, respectively. For example, at 800 K, the calculated CVT/SCT rate constant was 1.6 s^−1^ for the rate-determining step in the pathways 1–2, whereas the value was 3.6 × 10 ^−1^ s^−1^ for the rate-determining step in pathways 3–4 for the formation of DBF and methyl-DBF from BF with CPD. At 1000 K, the calculated CVT/SCT rate constant was 6.6 × 10^3^ s^−1^ for the rate-determining step in pathways 5–6, whereas the value was 4.7 × 10^3^ s^−1^ for the rate-determining step in pathways 7–8 for the formation of DBT and methyl-DBT from BT with CPDyl. Similarly, at 1200 K, the calculated CVT/SCT rate constant in pathways 9–10 was 7.4 × 10^4^ s^−1^, which was larger than that in pathways 11–12 (4.0 × 10^4^ s^−1^) for the formation of CA and methyl-CA from IN with CPDyl. All these comparisons agree well with the conclusions outlined above.

For the formation of dibenzo-NSO-HET from the reactions of benzo-NSO-HETs with the CPDyl, pathways 1, 5, and 9 were favored over pathways 2, 6, and 10, respectively; pathways 3, 7, and 11 were competitive with pathways 4, 8, and 12, respectively. This conclusion was also supported by comparing the CVT/SCT rate constants of the last two steps of these pathways. For example, for the formation of DBT and methyl-DBT in pathway 5–6, the calculated CVT/SCT rate constants was 1.1 × 10^6^ s^−1^ for the reaction IM17 → IM19 via TS25 at 1000 K, which was smaller than the value 1.8 × 10^7^ s^−1^ for the IM17 → IM20 via TS27 at 1000 K; the calculated CVT/SCT rate constant was 7.4 × 10^7^ s^−1^ for the reaction IM19 → dibenzothiophene + CH_3_ via TS26 at 1000 K, which was also smaller than the value 9.1 × 10^7^ s^−1^ for the IM20 → 1-methyl-dibenzothiophene + H via TS28 at 1000 K. This reconfirms the finding of the thermodynamic analysis: that pathway 6 resulting in 1-methyl-DBT was energetically favored over pathway 5 resulting in the DBT. For the formation of DBT and 4-methyl-DBT in pathway 7–8 at 800 K, the calculated CVT/SCT rate constant of IM18 → IM21 via TS29 was 3.3 × 10^5^ s^−1^, which was larger than the value 1.6 × 10^4^ s^−1^ for IM18 → IM22 via TS31; the value of IM21 → dibenzothiophene + CH_3_ via TS30 was 2.6 × 10^6^ s^−1^, which was smaller than the value 2.8 × 10^7^ s^−1^ of IM22 → 4-methyl-dibenzothiophene + H via TS32. This reconfirmed the thermodynamic analysis that both DBT and 4-methyl-DBT can be formed competitively.

For the pathways from the first C11–H shift or from the first C10–H shift in Figure 2, Figure 3 and Figure 4, the CVT/SCT rate constants for the rate-determining step of O heterocycle pathways, S heterocycle pathways, and N heterocycle pathways were ranked as S heterocycle pathways > N heterocycle pathways > O heterocycle pathways over the whole studied temperature range. For example, at 1000 K, for the pathways from the first C11–H shift of reactions of BF, BT, and IN with CPD, the CVT/SCT rate constants for the rate-determining step were 8.1 × 10^2^ s^−1^, 6.6 × 10^3^ s^−1^, and 1.2 × 10^3^ s^−1^ for the reactions of BF, BT, and IN with CPDyl, respectively; the value was 9.7 × 10^1^ s^−1^, 4.7 × 10^3^ s^−1^, and 6.0 × 10^2^ s^−1^ for the pathways from the first C11–H shift of reactions of BF, BT, and IN with CPDyl, respectively. This was consistent with the thermodynamic analysis: the ranking of the dibenzo-NSO-HET formation potential was sulfureted heterocycle compounds > nitrided heterocycle compounds > oxygenated heterocycle compounds.

## 4. Materials and Methods

### 4.1. Density Functional Theory

All the quantum chemical calculations on the structure, frequency, and energy of related substances such as reactants, products, intermediates, and transition state were performed using the Gaussian 09 program [70]. The geometry optimizations were conducted using the hybrid meta functional MPWB1K, which gives uniformly excellent performance for thermochemistry, thermochemical kinetics, hydrogen bonding, and weak interactions, with the standard 6-31+G(d,p) basis set [71]. The vibrational frequencies were also computed at the same level to determine the natures of the stationary points. Moreover, the intrinsic reaction coordinate (IRC) was calculated at the MPWB1K/6-31+G(d,p) level to verify that the transition state connected to the right minima along the reaction path [72]. In order to acquire more reliable energy information, a more flexible basis set, 6-311+G(3df,2p), was employed to determine the single point energies. All energies quoted and discussed in this paper include zero-point energy correction (ZPE).

### 4.2. Kinetic Calculation

To obtain the rate constants and activation energies, kinetic calculations were carried out the using POLYRATE 9.7 program [63]. The canonical variational transition state (CVT) theory with a small-curvature tunneling (SCT) contribution [64,66,67] was applied to evaluate the theoretical rate constants for the elementary steps included in the reactions of BF, BT, and IN with CPDyl. The rate constants for each reaction were calculated over a wide temperature range (600–1200 K), which covered the possible DBF, DBT, and CA formation temperatures under pyrolysis or combustion conditions. To calculate the rate constants, 40 nonstationary points near the transition state along the MEP (20 points on the reactant side and 20 points on the product side) were selected for frequency calculations at the MPWB1K/6-31+G(d,p) level. The SSTEP, a variable keyword in the POLYRATE 9.7 program that specifies the step size along the mass-scaled MEP, was confirmed as 0.05. The SRANGE, which is required to specify the limits on the reaction coordinates, was selected as −1.5 to 1.5. Parameters such as energy data, force constant matrices, hessian matrices, stationary point coordinates, and unstationary points were obtained from the Gaussian 09 program output files and were input into the POLYRATE 9.7 input files automatically by our self-compiled program.

## 5. Conclusions

In this study, the mechanisms of the homogeneous gas-phase formation of dibenzo-NSO-heterocycles (dibenzo-NSO-HETs), namely dibenzofuran (DBF), dibenzothiophene (DBT), and carbazole (CA), from benzo-NSO-heterocycles (benzo-NSO-HETs), namely benzofuran (BF), benzothiophene (BT), and indole (IN), with the cyclopentadienyl radical (CPDyl) were investigated theoretically using DFT electronic structure theory at the MPWB1K/6-311+G(3df,2p)//MPWB1K/6-31+G(d,p) level. Kinetic calculations were performed and the rate constants were calculated over the temperature range of 600−1200 K using canonical variational transition state (CVT) theory with the small-curvature tunneling (SCT) contribution. The rate temperature formulas were fitted. Several energetically preferred routes for dibenzo-NSO-HET formation were proposed. The obtained rate constant can support more detailed input parameters for the NSO-HETs control and prediction models. The effects of O, S, and N atoms on the formation potential for dibenzo-NSO-HET formation were sorted. Four specific conclusions were drawn.

(1) The dibenzo-NSO-HETs growth mechanism involves six elementary steps: the addition reaction, ring closure, the first H shift, C–C cleavage, the second H shift, and elimination of CH_3_ or H. Due to its having the highest activation barrier and being strongly endothermic, the cleavage of the C–C bond step was regarded as the rate-determining step for each pathway.

(2) For given dibenzo-NSO-HET formation, pathways from an initial C11–H shift step were preferred over pathways from an initial C10–H shift step. The formation of C1-methylated products was favored over that of C4-methylated products. DBF and 1-methyl-DBF, DBT and 1-methyl-DBT, and CA and 1-methyl-CA were the main products of reactions of BF, BT, and IN with CPDyl, respectively.

(3) The ranking of the dibenzo-NSO-HET formation potential from benzo-NSO-HETs with CPDyl was as follows: sulfureted heterocycle compounds > nitrided heterocycle compounds > oxygenated heterocycle compounds. The reactions of CPDyl attacking the benzene and furan/thiophene/pyrrole rings were inferred to be comparable under pyrolysis or combustion conditions.

## Figures and Tables

**Table 1:** Arrhenius formulas for dibenzofuran (DBF) formation routes from the reaction of benzofuran (BF) with cyclopentadienyl radical (CPDyl) over the temperature range of 600−1200 K (units are s^−1^ and cm^3^ molecule^−1^ s^−1^ for unimolecular and bimolecular reactions, respectively).

Reactions	Arrhenius Formulas
benzofuran + cyclopentadieny → IM1 via TS1	*k*(T) = (10.0 × 10^−25^) exp (−12066.6/T)
benzofuran + cyclopentadieny → IM1 via TS2	*k*(T) = (1.2 × 10^−25^) exp (−13171.4/T)
IM1 → IM3 via TS3	*k*(T) = (3.0 × 10^11^) exp (−5026.9/T)
IM2 → IM3 via TS4	*k*(T) = (2.6 × 10^13^) exp (−10547.3/T)
IM3 → IM4 via TS5	*k*(T) = (9.2 × 10^12^) exp (−23362.2/T)
IM3 → IM5 via TS6	*k*(T) = (1.0 × 10^13^) exp (−23976.6/T)
IM4 → IM6 via TS7	*k*(T) = (4.9 × 10^13^) exp (−24826.0/T)
IM5 → IM7 via TS8	*k*(T) = (5.1 × 10^11^) exp (−22376.3/T)
IM6 → IM8 via TS9	*k*(T) = (7.0 × 10^12^) exp (−15749.0/T)
IM8 → dibenzofuran + CH_3_ via TS10	*k*(T) = (5.2 × 10^13^) exp (−12901.2/T)
IM6 → IM9 via TS11	*k*(T) = (8.4 × 10^12^) exp (−15584.3/T)
IM9 → 4-methyl-dibenzofuran + H via TS12	*k*(T) = (1.6 × 10^13^) exp (−11575.9/T)
IM7 → IM10 via TS13	*k*(T) = (2.7 × 10^12^) exp (−13593.4/T)
IM10 → dibenzofuran + CH_3_ via TS14	*k*(T) = (6.8 × 10^13^) exp (−13629.3/T)
IM7 → IM11 via TS15	*k*(T) = (7.8 × 10^12^) exp (−13970.9/T)
IM11 → 2-methyl-dibenzofuran + H via TS16	*k*(T) = (2.5 × 10^13^) exp (−10298.0/T)

**Table 2:** Arrhenius formulas for dibenzothiophene (DBT) formation routes from the reaction of benzothiophene (BT) with cyclopentadienyl radical (CPDyl) over the temperature range of 600−1200 K (units are s^−1^ and cm^3^ molecule^−1^ s^−1^ for unimolecular and bimolecular reactions, respectively).

Reactions	Arrhenius Formulas
benzothiophene + cyclopentadieny → IM12 via TS17	*k*(T) = (3.0 × 10^−25^) exp (−10457.6/T)
benzothiophene + cyclopentadieny → IM13 via TS18	*k*(T) = (2.1 × 10^−24^) exp (−10618.3/T)
IM12 → IM14 via TS19	*k*(T) = (3.3 × 10^11^) exp (−14334.0/T)
IM13 → IM14 via TS20	*k*(T) = (2.6 × 10^11^) exp (−12339.6/T)
IM14 → IM15 via TS21	*k*(T) = (1.7 × 10^13^) exp (−21731.2/T)
IM14 → IM16 via TS22	*k*(T) = (1.5 × 10^13^) exp (−21514.7/T)
IM15 → IM17 via TS23	*k*(T) = (9.8 × 10^13^) exp (−23422.3/T)
IM16 → IM18 via TS24	*k*(T) = (1.2 × 10^14^) exp (−23936.3/T)
IM17 → IM19 via TS25	*k*(T) = (1.8 × 10^12^) exp (−14289.8/T)
IM19 → dibenzothiophene + CH_3_ via TS26	*k*(T) = (5.2 × 10^13^) exp (−13461.3/T)
IM17 → IM20 via TS27	*k*(T) = (5.8 × 10^12^) exp (−12699.8/T)
IM20 → 4-methyl-dibenzothiophene + H via TS28	*k*(T) = (2.5× 10^13^) exp (−12510.8/T)
IM18 → IM21 via TS29	*k*(T) = (2.8 × 10^13^) exp (−12756.2/T)
IM21 → dibenzothiophene + CH_3_ via TS30	*k*(T) = (8.9 × 10^13^) exp (−13857.5/T)
IM18 → IM22 via TS31	*k*(T) = (6.8 × 10^12^) exp (−15889.8/T)
IM22 → 2-methyl-dibenzothiophene + H via TS32	*k*(T) = (1.7 × 10^13^) exp (−10615.2/T)

**Table 3:** Arrhenius formulas for carbazole (CA) formation routes from the reaction of indole (IN) with cyclopentadienyl radical (CPDyl) over the temperature range of 600−1200 K (units are s^−1^ and cm^3^ molecule^−1^ s^−1^ for unimolecular and bimolecular reactions, respectively).

Reactions	Arrhenius Formulas
indole + cyclopentadieny → IM23 via TS33	*k*(T) = (1.1 × 10^−24^) exp (−11654.9/T)
indole + cyclopentadieny → IM24 via TS34	*k*(T) = (1.8 × 10^−24^) exp (−8209.2/T)
IM23 → IM25 via TS35	*k*(T) = (5.8 × 10^10^) exp (−11877.8/T)
IM24 → IM25 via TS36	*k*(T) = (2.0 × 10^11^) exp (−14906.8/T)
IM25 → IM26 via TS37	*k*(T) = (1.6 × 10^13^) exp (−22274.5/T)
IM25 → IM27 via TS38	*k*(T) = (1.5 × 10^13^) exp (−21960.5/T)
IM26 → IM28 via TS39	*k*(T) = (4.3 × 10^13^) exp (−24215.9/T)
IM27 → IM29 via TS40	*k*(T) = (4.5 × 10^13^) exp (−25026.4/T)
IM28 → IM30 via TS41	*k*(T) = (6.2 × 10^10^) exp (−10484.5/T)
IM30 → carbazole + CH_3_ via TS42	*k*(T) = (8.1 × 10^13^) exp (−13792.3/T)
IM28 → IM31 via TS43	*k*(T) = (3.7 × 10^12^) exp (−11906.8/T)
IM31 → 4-methyl-carbazole + H via TS44	*k*(T) = (2.3 × 10^13^) exp (−10279.5/T)
IM29 → IM32 via TS45	*k*(T) = (2.0 × 10^12^) exp (−13357.4/T)
IM32 → carbazole + CH_3_ via TS46	*k*(T) = (7.6 × 10^13^) exp (−13643.4/T)
IM29 → IM33 via TS47	*k*(T) = (6.4 × 10^12^) exp (−15841.1/T)
IM33 → 2-methyl-carbazole + H via TS48	*k*(T) = (2.7 × 10^13^) exp (−10075.0/T)

## Supplementary Materials

The following are available online at https://www.mdpi.com/1422-0067/20/21/5420/s1.

## Abbreviations

DBF	dibenzofuran
DBT	dibenzothiophene
CA	carbazole
BF	benzofuran
BT	benzothiophene
IN	indole
NSO-HETs	nitrogen, sulfur, or oxygen heterocycles
PACs	polycyclic aromatic compounds
PAHs	polycyclic aromatic hydrocarbons
DFT	density functional theory
CVT	canonical variational transition state
SCT	small-curvature tunneling
CPDyl	cyclopentadienyl radical
ZPE	zero-point energy
IRC	intrinsic reaction coordinate
