# Supplementary material for: The Gas-Phase Formation Mechanism of Dibenzofuran (DBF), Dibenzothiophene (DBT), and Carbazole (CA) from Benzofuran (BF), Benzothiophene (BT), and Indole (IN) with Cyclopentadienyl Radical
Source: Int J Mol Sci. 2019 Oct 31;20(21):5420. doi: 10.3390/ijms20215420 (PMC6861977; doi:10.3390/ijms20215420)
Supplement: Supplementary file 1 [file ijms-20-05420-s001.pdf]

Supporting Information for

The Gas-phase Formation Mechanism of Dibenzofuran(DBF), Dibenzothiophene(DBT) and Carbazole(CA) from Benzofuran (BF), Benzothiophene (BT) and Indole (IN) with Cyclopentadienyl Radical

Xuan Li[1,†], Yixiang Gao[1,†], Chenpeng Zuo[1,2], Siyuan Zheng[1], Fei Xu[1,2] *, Yanhui Sun[3] , Qingzhu Zhang[1]

[1] Environment Research Institute, Shandong University, Qingdao 266237, P. R. China; lx18265450270@126.com, yixianggao@163.com zuochenpeng@126.com, Kishi_Wang@163.com, zhengsiyuan1991@126.com, xufei@sdu.edu.cn, zqz@sdu.edu.cn

[2] Shenzhen Research Institute, Shandong University, Shenzhen 518057, P. R. China;

[3] College of Environment and Safety Engineering, Qingdao University of Science & Technology, Qingdao 266042, P. R. China; sunyh0532@126.com

* Correspondence: xufei@sdu.edu.cn; Tel.: +86-532-58631992

† These authors contributed equally to this article

Received: date; Accepted: date; Published: date

Keywords: NSO-HETs; Formation mechanism; Cyclopentadienyl; Rate constant; Density functional method

Sixty eight pages

Contains three figures and eight tables

Table S1. Imaginary frequencies (in cm^{-1}), total energies (in a.u.), zero point energies (ZPE, in a.u.), thermal correction to energy (in a.u.), thermal correction to enthalpy (in a.u.), and thermal correction to gibbs free energy (in a.u.) for the transition states, intermediates, reactions, and products involved in this paper.

Table S2. Relative activation barriers ΔE_r (in kcal/mol) and relative reaction heats ΔH_r (in kcal/mol) in the reaction of NSO-HETs from the reactions of benzofuran, dibenzothiophene and indole with the cyclopentadienyl radicals. ΔH is calculated at 0 K.

Figure S1. The structures embedded with the geometrical parameters for the transition states in the reaction of benzofuran with cyclopentadienyl radical. Distances are in angstrom.

Figure S2. The structures embedded with the geometrical parameters of transition states in benzothiophene with cyclopentadienyl radical. Distances are in angstrom.

Figure S3. The structures embedded with the geometrical parameters for transition states in the reaction of indole with cyclopentadienyl radical. Distances are in angstrom.

Table S3. The observed and calculated vibrational frequencies of benzofuran, benzothiophene and indole (in cm^{-1}).

Table S4. The observed and calculated vibrational frequencies of dibenzofuran, dibenzothiophene, and carbazole (in cm^{-1}).

Table S5. The observed and calculated bond distances (A) in benzofuran, indole, dibenzofuran, dibenzothiophene, and carbazole.

Table S6. CVT/SCT rate constants for crucial elementary reactions involved in the formation of NSO-HETs from the reactions of benzofuran, dibenzothiophene, and indole with the cyclopentadienyl radicals over the temperature range of 600-1200 K (units are s^{-1} and cm^3 $molecule^{-1}$ s^{-1} for unimolecular and bimolecular reactions, respectively).

Table S7. Cartesian coordinates for the transition states involved in the NSO-HETs formation routes, x coordinate, y coordinate, and z coordinate.

Table S8. Cartesian coordinates for reactions, intermediate and products involved in the NSO-HETs formation routes, x coordinate, y coordinate and z coordinate.

Table S1. Imaginary frequencies (in cm^{-1}), total energies (in a.u.), zero point energies (ZPE, in a.u.), thermal correction to energy (in a.u.), thermal correction to enthalpy (in a.u.), and thermal correction to gibbs free energy (in a.u.) for the transition states, intermediates, reactions, and products involved in this paper.

Transition states	Imaginary frequencies	Total energies	ZPE	Thermal correction to Energy	Thermal correction to Enthalpy	Thermal correction to Gibbs Free Energy
TS1	-596i	-576.803777	0.204367	0.214793	0.215737	0.166456
TS2	-618i	-576.796351	0.204564	0.214841	0.215785	0.167529
TS3	-585i	-576.788658	0.206207	0.215459	0.216403	0.170808
TS4	-512i	-576.780408	0.206018	0.246782	0.216295	0.170564
TS5	-2275i	-576.742220	0.204321	0.213270	0.214214	0.169283
TS6	-2282i	-576.739057	0.204046	0.213003	0.213947	0.168990
TS7	-633i	-576.755612	0.204751	0.214281	0.215226	0.169203
TS8	-709i	-576.744088	0.204581	0.214125	0.215069	0.169002
TS9	-2136i	-576.771930	0.201259	0.211341	0.212285	0.165017
TS10	-540i	-576.836351	0.202972	0.213945	0.214889	0.165408
TS11	-1804i	-576.772374	0.201789	0.212091	0.213035	0.165108
TS12	-991i	-576.829950	0.199671	0.210191	0.211135	0.163178
TS13	-2063i	-576.773060	0.201233	0.211280	0.212224	0.165166
TS14	-515i	-576.837721	0.203019	0.213911	0.214856	0.165708
TS15	-1887i	-576.772130	0.201105	0.211476	0.212421	0.164535
TS16	-1080i	-576.827213	0.199712	0.210057	0.211002	0.163593
TS17	-615i	-899.856584	0.200748	0.211774	0.212718	0.161879
TS18	-597i	-899.855537	0.201312	0.212135	0.213079	0.163572
TS19	-597i	-899.838841	0.202947	0.212812	0.213756	0.166648
TS20	-564i	-899.835683	0.203017	0.212872	0.213816	0.166840
TS21	-2259i	-899.797181	0.201064	0.210632	0.211576	0.165086
TS22	-2196i	-899.797856	0.201119	0.210671	0.211615	0.165191
TS23	-631i	-899.815684	0.201726	0.211872	0.212817	0.165189
TS24	-642i	-899.808676	0.201798	0.211921	0.212865	0.165308
TS25	-2082i	-899.827471	0.198363	0.209030	0.209975	0.161182
TS26	-535i	-899.832418	0.199833	0.211391	0.212335	0.161451
TS27	-1875i	-899.828282	0.198402	0.209263	0.210207	0.161066
TS28	-1068i	-899.818429	0.196431	0.207470	0.208414	0.159254
TS29	-2013i	-899.890716	0.198245	0.208885	0.209829	0.161260
TS30	-512i	-899.882381	0.199865	0.211371	0.212315	0.161691
TS31	-1854i	-899.891954	0.198555	0.209380	0.210324	0.161412
TS32	-1070i	-899.878758	0.196725	0.207623	0.208567	0.159717
TS33	-569i	-556.925132	0.216996	0.227718	0.228662	0.179470
TS34	-523i	-556.934279	0.217496	0.227941	0.228885	0.180181
TS35	-508i	-556.903208	0.218507	0.228261	0.229205	0.182790
TS36	-590i	-556.904143	0.218967	0.228454	0.229398	0.183498
TS37	-2268i	-556.860368	0.217144	0.226283	0.227227	0.182040
TS38	-2255i	-556.860797	0.216831	0.226020	0.226964	0.181659
TS39	-636i	-556.876365	0.217688	0.227373	0.228318	0.182086
TS40	-691i	-556.871195	0.217655	0.227369	0.228313	0.182025
TS41	-2088i	-556.893516	0.213965	0.224298	0.225243	0.177607
TS42	-543i	-556.961655	0.215572	0.226916	0.227861	0.177839
TS43	-1861i	-556.898210	0.214464	0.224907	0.225851	0.178022
TS44	-998i	-556.954122	0.212430	0.223192	0.224136	0.175895
TS45	-2073i	-556.897910	0.213882	0.224252	0.225196	0.177651
TS46	-518i	-556.963082	0.215567	0.226857	0.227802	0.178031
TS47	-1876i	-556.889918	0.214062	0.224669	0.225613	0.177581
TS48	-1057i	-556.953209	0.212481	0.223197	0.224141	0.176134

Reactions, intermediate and products	Total energies	ZPE	Thermal correction to Energy	Thermal correction to Enthalpy	Thermal correction to Gibbs Free Energy
IM1	-576.8048879	0.206064	0.216528	0.217472	0.168263
IM2	-576.8138617	0.206259	0.216818	0.217762	0.168581
IM3	-576.8139706	0.209111	0.218306	0.219250	0.173848
IM4	-576.8307990	0.209683	0.218800	0.219745	0.174423
IM5	-576.8248745	0.209952	0.218982	0.219926	0.174848
IM6	-576.8176478	0.204981	0.215989	0.216933	0.167022
IM7	-576.8149233	0.205072	0.215850	0.216794	0.168117
IM8	-576.8761111	0.207277	0.217704	0.218648	0.170591
IM9	-576.8655279	0.206641	0.217080	0.218024	0.169969
IM10	-576.8789877	0.207133	0.217531	0.218475	0.170604
IM11	-576.8578121	0.206601	0.216887	0.217831	0.170215
IM12	-899.8843251	0.202838	0.213856	0.214801	0.164205
IM13	-899.8750020	0.202897	0.214040	0.214985	0.164310
IM14	-899.8642949	0.206003	0.215757	0.216701	0.169925
IM15	-899.8867526	0.206230	0.215986	0.216931	0.170026
IM16	-899.8811503	0.206726	0.216385	0.21733	0.170671
IM17	-899.8714025	0.202065	0.213479	0.214423	0.164052
IM18	-899.8676349	0.202319	0.213661	0.214605	0.164508
IM19	-899.9310743	0.204069	0.215071	0.216016	0.166446
IM20	-899.9193908	0.203365	0.214438	0.215382	0.16568
IM21	-899.9324310	0.203946	0.214922	0.215867	0.166599
IM22	-899.9099042	0.203577	0.214470	0.215414	0.165885
IM23	-556.9406759	0.218865	0.229722	0.230666	0.181242
IM24	-556.9514257	0.218582	0.229374	0.230318	0.180703
IM25	-556.9283275	0.221725	0.231038	0.231982	0.186508
IM26	-556.9505155	0.222233	0.231585	0.232529	0.18693
IM27	-556.9473903	0.222335	0.231828	0.232773	0.186867
IM28	-556.9362276	0.218165	0.229206	0.23015	0.18107
IM29	-556.9392348	0.217947	0.229027	0.229971	0.180899
IM30	-557.0023212	0.219902	0.230678	0.231622	0.182968
IM31	-556.9842207	0.219308	0.229964	0.230909	0.182501
IM32	-557.0041437	0.219847	0.230609	0.231553	0.183121
IM33	-556.9828758	0.219253	0.229880	0.230825	0.182634
benzofuran	-383.4891292	0.120984	0.126808	0.127752	0.090943
benzothiophene	-706.5454749	0.117504	0.123849	0.124793	0.086572
indole	-363.6167017	0.133670	0.139807	0.140752	0.103468
cyclopentadienyl	-193.3363894	0.080056	0.083938	0.084882	0.053066
dibenzofuran	-537.0662141	0.169360	0.177670	0.178614	0.135829
dibenzothiophene	-860.1197136	0.166121	0.175048	0.175993	0.131667
carbazole	-517.1915252	0.182029	0.190705	0.191649	0.148287
4-methyl-dibenzofuran	-576.3444811	0.196817	0.207132	0.208076	0.160298
1-methyl-dibenzofuran	-576.3438980	0.197667	0.207704	0.208649	0.162116
4-methyl-dibenzothiophene	-899.3981728	0.194469	0.205012	0.205956	0.158295
1-methyl-dibenzothiophene	-899.3941169	0.194922	0.205259	0.206203	0.158994
1-methyl-carbazole	-556.4684220	0.210519	0.220722	0.221666	0.175174
4-methyl-carbazole	-556.4691416	0.220561	0.221505	0.210085	0.174199
cyclopentadiene	-193.959256	0.095124	0.099211	0.100156	0.068566
methane	-40.449572	0.045849	0.048714	0.049658	0.028547

Table S2. Relative activation barriers ΔE_r (in kcal/mol) and relative reaction heats ΔH_r (in kcal/mol) in the reaction of NSO-HETs from the reactions of benzofuran, dibenzothiophene and indole with the cyclopentadienyl radicals. ΔH is calculated at 0 K.

Transition states	ΔE_r	ΔH_r
TS1	13.64	12.94
TS2	18.29	7.31
TS3	23.12	7.24
TS4	28.29	7.24
TS5	52.25	-3.31
TS6	54.23	0.40
TS7	43.85	4.94
TS8	51.07	6.65
TS9	33.61	-31.73
TS10	-6.79	-17.97
TS11	33.33	-25.09
TS12	-2.78	-10.16
TS13	32.90	-33.54
TS14	-7.65	-17.97
TS15	33.49	-20.25
TS16	-1.06	-10.53
TS17	15.86	-1.54
TS18	16.51	4.30
TS19	26.98	11.02
TS20	28.97	11.02
TS21	53.11	-3.07
TS22	52.69	0.45
TS23	41.51	6.56
TS24	45.90	8.92
TS25	34.12	-30.87
TS26	-5.55	-16.18
TS27	31.01	-23.54
TS28	-0.32	-8.86
TS29	33.61	-31.72
TS30	-6.33	-16.18
TS31	39.79	-17.59
TS32	1.95	-6.32
TS33	17.54	7.79
TS34	11.80	1.04
TS35	31.29	15.53
TS36	30.70	15.53
TS37	58.16	1.62
TS38	57.89	3.58
TS39	48.12	10.58
TS40	51.37	8.69
TS41	37.37	-30.88
TS42	-5.37	-16.55
TS43	34.42	-19.52
TS44	-0.65	-8.25
TS45	34.61	-32.02
TS46	-6.27	-16.55
TS47	39.62	-18.68
TS48	-0.07	-8.70

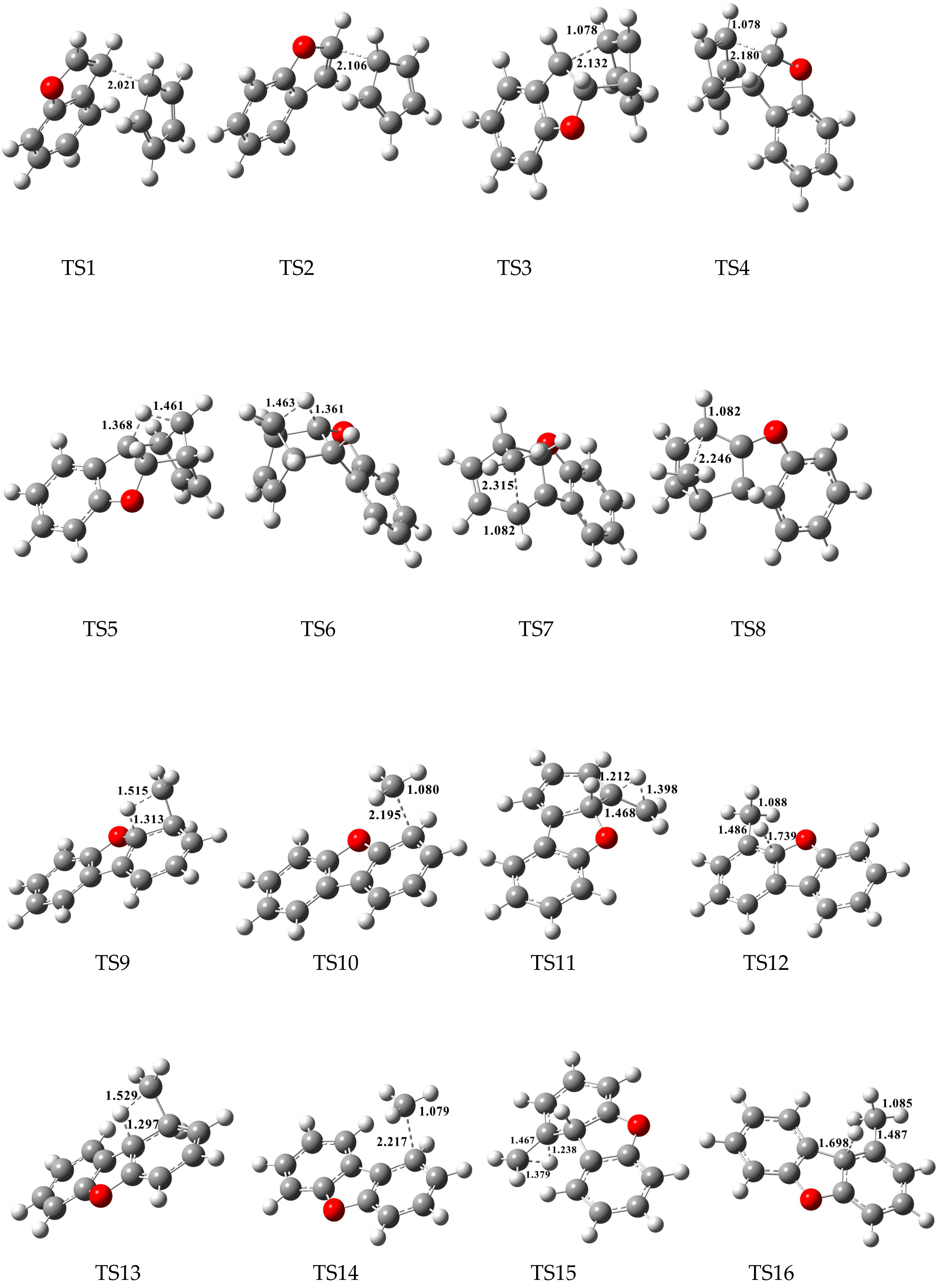
2.021
TS1
2.106
TS2
1.078
2.132
TS3
1.078
2.180
TS4
1.461
1.368
TS5
1.463
1.361
TS6
2.315
1.082
TS7
1.082
2.246
TS8
1.515
1.313
TS9
1.080
2.195
TS10
1.212
1.398
1.468
TS11
1.088
1.486
1.739
TS12
1.529
1.297
TS13
1.079
2.217
TS14
1.467
1.238
1.379
TS15
1.085
1.698
1.487
TS16

Figure S1

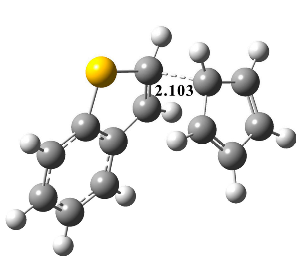

TS17

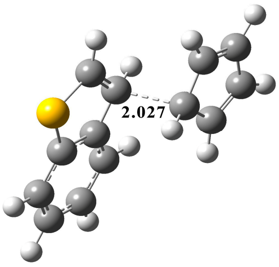

TS18

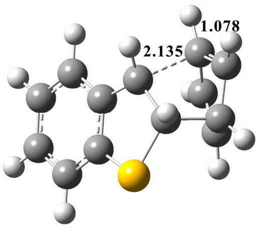

TS19

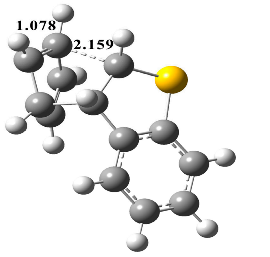

TS20

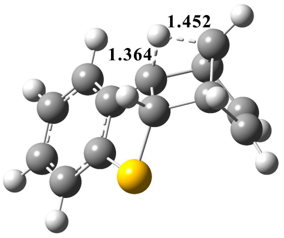

TS21

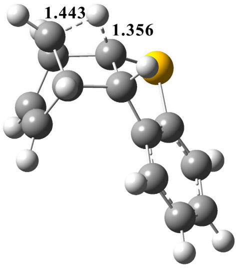

TS22

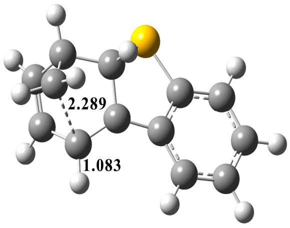

TS23

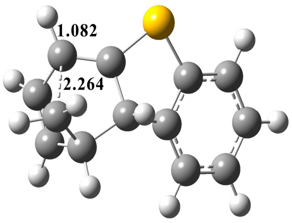

TS24

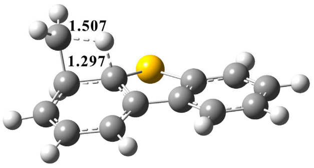

TS25

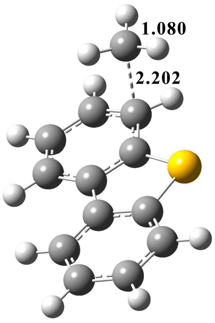

TS26

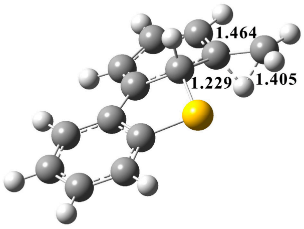

TS27

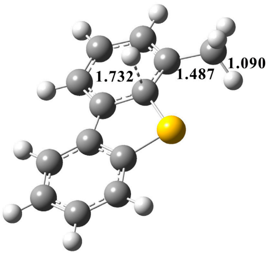

TS28

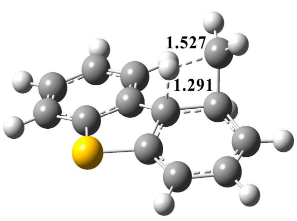

TS29

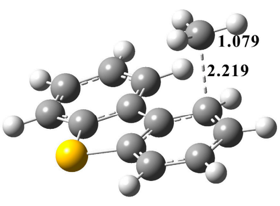

TS30

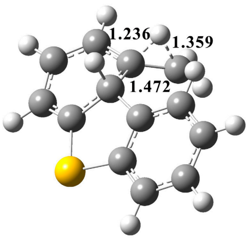

TS31

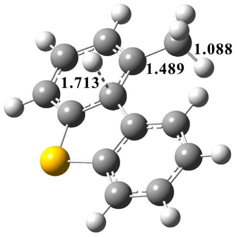

TS32

Figure S2

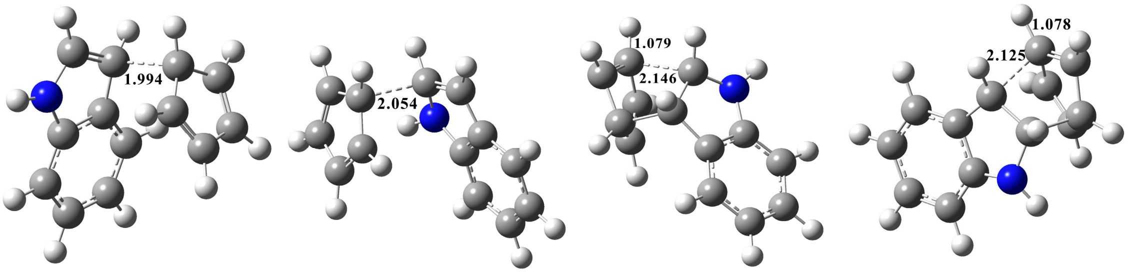

TS33 TS34 TS35 TS36

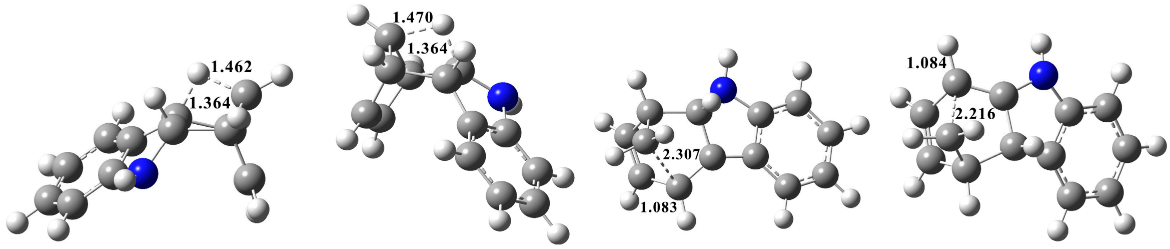

TS37 TS38 TS39 TS40

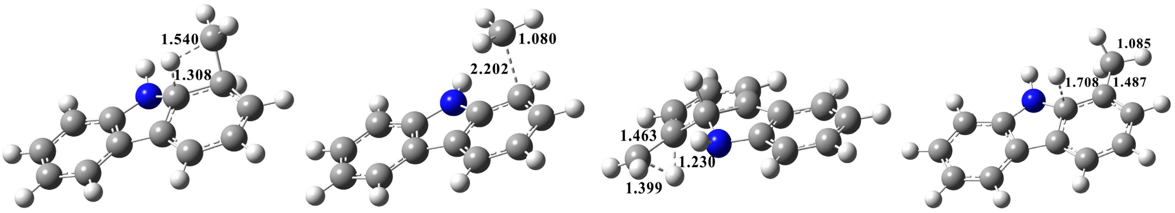

TS41 TS42 TS43 TS44

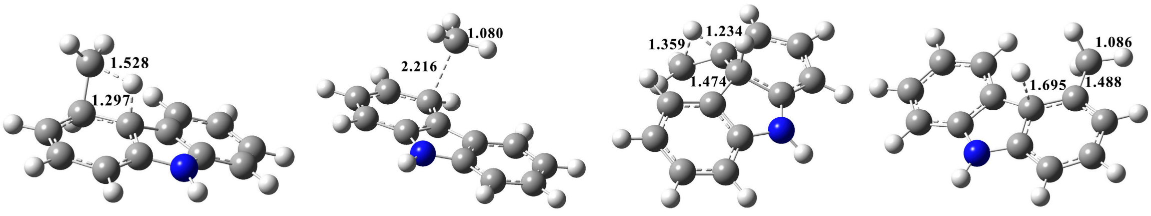

TS45 TS46 TS47 TS48

Figure S3

Table S3. The observed and calculated vibrational frequencies of benzofuran, benzothiophene, and indole (in cm^{-1}).

Benzofuran		Benzothiophene		Indole	
Calculated	Observed[a]	Calculated	Observed[a]	Calculated	Observed[a]
3377	3158	3340	3130	3802	3524
3351	3125	3307	3116	3360	3140
3307	3094	3298	3087	3340	3119
3295	3077	3289	3076	3295	
3284	3067	3278	3071	3284	3072
3273	3049	3270	3061	3273	3058
1737	1617	1717	1597	3266	3051
1710	1595	1681	1567	1740	1625
1653	1543	1617	1502	1696	1580
1559	1479	1543	1461	1621	1519
1541	1457	1507	1425	1580	1486
1443	1346	1420	1345	1542	1458
1409	1329	1405	1318	1508	1411
1346	1264	1312	1260	1430	1347
1322	1253	1265	1209	1422	1333
1247	1179	1206	1162	1349	1276
1208	1164	1185	1135	1298	1245
1192	1131	1141	1092	1262	1205
1157	1107	1099	1058	1195	1150
1106	1036	1077	1016	1179	1121
1065	1009	1028	996	1146	1093
1022	968	990	936	1119	1067
984	929	942	881	1068	1014
932	900	900	869	1017	961
919	861	895	850	978	925
894	855	843	801	929	899
891	847	799	762	912	877
805	767	769	735	900	850
800	763	737	711	887	840
784	746	724	690	802	764
764	732	692	669	794	758
629	612	582	558	776	738
613	584	542	526	754	715
595	569	506	491	631	607
556	539	489	471	624	601
439	418	430	411	597	570
416	401	351	340	558	543
257	246	207	198	442	419
224	211	198	192	426	397
				407	387
				251	241
				221	207

[a] The observed value for vibrational frequencies taken from reference (Collier and Klots 1995).

Table S4. The observed and calculated vibrational frequencies of dibenzofuran, dibenzothiophene, and carbazole (in cm^{-1}).

Dibenzofuran		Dibenzothiophene		Carbazole	
Calculated	Observed[a]	Calculated	Observed[b]	Calculated	Observed[c]
3305	3265	3300	3117	3801	3421
3305	3194	3299	3065	3295	3094
3295		3291	3060	3295	3084
3295		3289	3055	3282	3077
3281		3281	3055	3282	3055
3281		3278	3025	3271	3050
3272	3081	3273	3025	3270	3039
3272		3269	3000	3265	3030
1760		1725	1601	3265	2940
1725		1710	1590	1752	
1713		1686	1566	1733	
1706	1640	1672	1558	1703	
1585	1633	1568	1480	1675	
1561		1541	1462	1595	1594
1537		1520	1442	1581	1576
1534	1489	1505	1421	1545	
1439	1446	1405	1334	1540	
1422		1399	1321	1481	1490
1374	1351	1392	1314	1424	1481
1345	1339	1321	1268	1410	1452
1328	1315	1297	1236	1382	1380
1290	1306	1210	1171	1360	1334
1256	1242	1209	1155	1308	1320
1204		1199	1137	1271	1288
1195	1190	1187		1270	1233
1168	1148	1138	1078	1204	1205
1154	1102	1115	1072	1196	1204
1074		1090	1027	1175	1158
1069		1081	1027	1162	1152
1048		1038	990	1078	1136
1024	1029	1028	973	1076	1118
1023	1010	1026		1043	1107
985		989	940	1019	1022
985		988	938	1018	1012
896		897		981	995
895		896	859	980	926
895	871	799	771	908	910
889	850	797	768	890	
790		778	740	890	880
788		752	730	887	856
779		739	704	799	835
765		736	704	788	747
753	745	733	704	777	741
684		631	612	774	737
634	660	588	562	760	722
594		516	509	680	658
590		514	498	637	
576	555	512	497	599	616
539	516	447	421	590	566
455		438	420	571	548
443	424	434	410	526	
440		423		461	
324	286	283	287	446	445
300		229	226	440	425
233	217	221	218	372	
156	202	137	140	303	310
111	104	102	107	301	299
				234	220
				154	139
				111	104

[a] The observed value for Vibrational frequencies taken from reference (Ljubic and Sabljic 2007)

[b] The observed value for Vibrational frequencies taken from reference (Lee 2001)

[c] The observed value for Vibrational frequencies taken from reference (Bree and Zwarich 2001)

Table S5. The observed and calculated bond distances (A) in benzofuran, indole, dibenzofuran, dibenzothiophene, and carbazole.

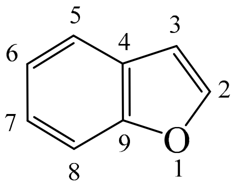

Atoms	Bond Distances	
	Calculated	Observed[a]
O_1-C_2	1.353	1.351
C_2-C_3	1.344	1.380
C_3-C_4	1.434	1.468
C_4-C_5	1.392	1.377
C_5-C_6	1.379	1.395
C_6-C_7	1.397	1.403
C_7-C_8	1.381	1.397
C_8-C_9	1.380	1.368
C_9-C_4	1.392	1.394
C_9-O_1	1.350	1.390

[a] The observed value for bond distances taken from reference (Maris et al., 2005)

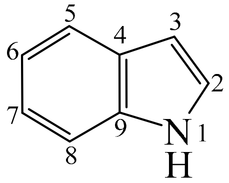

Atoms	Bond Distances	
	Calculated	Observed[b]
N_1-C_2	1.367	1.370
C_2-C_3	1.356	1.382
C_4-C_5	1.390	1.425
C_5-C_6	1.376	1.382
C_7-C_8	1.376	1.382
C_8-C_9	1.396	1.425
C_9-C_4	1.427	1.382
C_9-N	1.406	1.370

[b]The observed value for bond distances taken from reference (El-Azhary 1999)

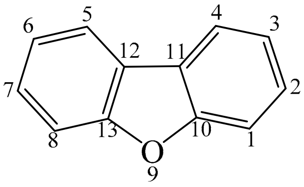

Atoms	Bond Distances	
	Calculated	Observed[c]
C_1-C_2	1.384	1.399
C_2-C_3	1.394	1.399
C_3-C_4	1.381	1.404
C_4-C_{11}	1.388	1.394
C_{11}-C_{12}	1.440	1.480
C_{11}-C_{10}	1.392	1.395
C_{10}-C_1	1.378	1.407
O_9-C_{10}	1.357	1.420

[c] The observed value for bond distances taken from reference (Banerjee 1972)

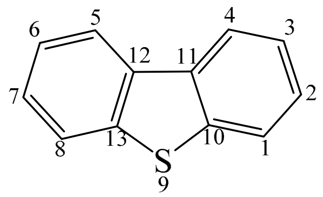

Atoms	Bond Distances	
	Calculated	Observed[d]
C_1-C_2	1.380	1.396
C_2-C_3	1.393	1.390
C_3-C_4	1.378	1.361
C_4-C_{11}	1.391	1.391
C_{11}-C_{12}	1.443	1.441
C_{11}-C_{10}	1.398	1.408
C_{10}-C_1	1.387	1.384
S_9-C_{10}	1.740	1.734

[d]The observed value for bond distances taken from reference (Schaffrin and Trotter 1970)

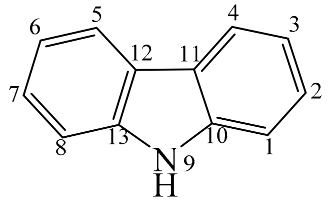

Atoms	Bond Lengths	
	Calculated	Observed[e]
C_1-C_2	1.380	1.390
C_2-C_3	1.395	1.398
C_3-C_4	1.380	1.395
C_4-C_{11}	1.389	1.400
C_{11}-C_{12}	1.439	1.467
C_{12}-C_{13}	1.404	1.404
C_{10}-C_1	1.366	1.395
C_5-H	1.079	0.897
C_6-H	1.079	0.981
C_7-H	1.079	1.070
C_8-H	1.080	0.983
N-H	1.371	1.414

[e]The observed value for bond distances taken from reference (Lee and Boo 1996)

Table S6. CVT/SCT rate constants for crucial elementary reactions involved in the formation of NSO-HETs from the reactions of benzofuran, dibenzothiophene, and indole with the cyclopentadienyl radicals over the temperature range of 600-1200 K (units are s^{-1} and cm^3 $molecule^{-1}$ s^{-1} for unimolecular and bimolecular reactions, respectively).

T(K)	CVT/SCT Rate Constants			
	TS1	TS2	TS3	TS4
600	2.1×10^{-33}	3.8×10^{-35}	6.8×10^{7}	6.0×10^{5}
700	3.1×10^{-32}	7.7×10^{-34}	2.3×10^{8}	7.2×10^{6}
800	2.6×10^{-31}	7.9×10^{-33}	5.5×10^{8}	4.9×10^{7}
900	1.4×10^{-30}	4.9×10^{-32}	1.1×10^{9}	2.1×10^{8}
1000	5.5×10^{-30}	2.2×10^{-31}	1.9×10^{9}	6.8×10^{8}
1100	1.8×10^{-29}	7.6×10^{-31}	3.1×10^{9}	1.8×10^{9}
1200	4.8×10^{-29}	2.2×10^{-30}	4.5×10^{9}	3.9×10^{9}
	TS5	TS6	TS7	TS8
600	1.2×10^{-5}	4.7×10^{-5}	5.4×10^{-5}	3.3×10^{-6}
700	2.9×10^{-2}	1.4×10^{-2}	1.9×10^{-2}	6.6×10^{-3}
800	1.9×10^{-1}	9.7×10^{-1}	1.6×10^{0}	3.5×10^{-1}
900	4.9×10^{1}	2.7×10^{1}	5.1×10^{1}	7.9×10^{0}
1000	6.5×10^{2}	4.0×10^{2}	8.1×10^{2}	9.6×10^{2}
1100	5.5×10^{3}	3.6×10^{3}	7.8×10^{3}	7.5×10^{3}
1200	3.3×10^{4}	2.3×10^{4}	5.2×10^{4}	4.2×10^{4}
	TS9	TS10	TS11	TS12
600	1.8×10^{1}	2.5×10^{4}	3.7×10^{1}	6.5×10^{4}
700	1.9×10^{3}	5.2×10^{5}	1.2×10^{3}	1.1×10^{6}
800	2.5×10^{4}	4.9×10^{6}	5.7×10^{4}	8.7×10^{6}
900	1.9×10^{5}	3.0×10^{7}	4.4×10^{5}	4.7×10^{7}
1000	9.8×10^{5}	1.3×10^{8}	1.3×10^{6}	1.4×10^{8}
1100	3.8×10^{6}	4.3×10^{8}	4.8×10^{6}	4.2×10^{8}
1200	1.2×10^{7}	1.2×10^{9}	1.5×10^{7}	1.0×10^{9}
	TS13	TS14	TS15	TS16
600	4.0×10^{2}	1.0×10^{4}	6.1×10^{2}	9.3×10^{5}
700	9.9×10^{3}	2.2×10^{5}	1.7×10^{4}	9.9×10^{6}
800	1.1×10^{5}	2.6×10^{6}	2.0×10^{5}	6.2×10^{7}
900	7.4×10^{5}	1.8×10^{7}	1.4×10^{6}	2.6×10^{8}
1000	3.4×10^{6}	8.2×10^{7}	6.7×10^{6}	8.4×10^{8}
1100	1.2×10^{7}	2.9×10^{8}	2.4×10^{7}	2.2×10^{9}
1200	3.3×10^{7}	8.3×10^{8}	7.0×10^{7}	4.9×10^{9}
	TS17	TS18	TS19	TS20
600	9.1×10^{-33}	4.8×10^{-32}	1.4×10^{1}	3.0×10^{2}
700	9.3×10^{-32}	5.1×10^{-31}	4.2×10^{2}	5.7×10^{3}
800	5.7×10^{-31}	3.2×10^{-30}	5.5×10^{3}	5.2×10^{4}
900	2.4×10^{-30}	1.4×10^{-29}	4.0×10^{4}	2.9×10^{5}
1000	8.2×10^{-30}	4.8×10^{-29}	2.0×10^{5}	1.1×10^{6}
1100	2.3×10^{-29}	1.4×10^{-28}	7.3×10^{5}	3.5×10^{6}
1200	5.6×10^{-29}	3.4×10^{-28}	2.2×10^{6}	8.9×10^{6}

T(K)	CVT/SCT Rate Constants			
	TS21	TS22	TS23	TS24
600	3.2×10^{-3}	4.0×10^{-3}	1.1×10^{-3}	5.6×10^{-4}
700	5.5×10^{-1}	6.5×10^{-1}	2.9×10^{-1}	1.6×10^{-1}
800	2.6×10^{1}	3.0×10^{1}	1.9×10^{2}	1.2×10^{1}
900	5.4×10^{2}	6.0×10^{2}	4.8×10^{2}	3.2×10^{2}
1000	6.0×10^{3}	6.6×10^{3}	6.6×10^{3}	4.7×10^{3}
1100	4.4×10^{4}	4.7×10^{4}	5.6×10^{4}	4.2×10^{4}
1200	2.3×10^{5}	2.4×10^{5}	3.3×10^{5}	2.6×10^{5}
	TS25	TS26	TS27	TS28
600	8.4×10^{1}	9.5×10^{3}	3.8×10^{3}	2.2×10^{4}
700	2.5×10^{3}	2.3×10^{5}	7.6×10^{4}	4.2×10^{5}
800	3.2×10^{4}	2.5×10^{6}	7.3×10^{5}	3.9×10^{6}
900	2.3×10^{5}	1.6×10^{7}	4.3×10^{6}	2.2×10^{7}
1000	1.1×10^{6}	7.4×10^{7}	1.8×10^{7}	9.0×10^{7}
1100	4.2×10^{6}	2.5×10^{8}	5.6×10^{7}	2.9×10^{8}
1200	1.2×10^{7}	7.1×10^{8}	1.5×10^{8}	7.5×10^{8}
	TS29	TS30	TS31	TS32
600	1.7×10^{3}	8.5×10^{3}	2.2×10^{1}	3.5×10^{5}
700	3.4×10^{4}	2.2×10^{5}	9.5×10^{2}	4.3×10^{6}
800	3.3×10^{5}	2.6×10^{6}	1.6×10^{4}	2.8×10^{7}
900	2.0×10^{6}	1.8×10^{7}	1.5×10^{5}	1.2×10^{8}
1000	8.1×10^{6}	8.5×10^{7}	8.5×10^{5}	4.1×10^{8}
1100	2.6×10^{7}	3.0×10^{8}	3.7×10^{6}	1.1×10^{9}
1200	6.9×10^{7}	8.8×10^{8}	1.2×10^{7}	2.5×10^{9}
	TS33	TS34	TS35	TS36
600	4.6×10^{-33}	2.3×10^{-30}	1.5×10^{2}	3.2×10^{0}
700	6.3×10^{-32}	1.4×10^{-29}	2.5×10^{3}	1.1×10^{2}
800	4.8×10^{-31}	5.5×10^{-29}	2.1×10^{4}	1.6×10^{3}
900	2.5×10^{-30}	1.8×10^{-28}	1.1×10^{5}	1.3×10^{4}
1000	9.4×10^{-30}	4.6×10^{-28}	4.1×10^{5}	6.6×10^{4}
1100	2.9×10^{-29}	1.0×10^{-27}	1.2×10^{6}	2.6×10^{5}
1200	7.6×10^{-29}	2.1×10^{-27}	3.0×10^{6}	8.0×10^{5}
	TS37	TS38	TS39	TS40
600	1.2×10^{-3}	1.9×10^{-3}	1.2×10^{-4}	3.5×10^{-5}
700	2.3×10^{-1}	3.4×10^{-1}	4.0×10^{-2}	1.3×10^{-2}
800	1.2×10^{1}	1.7×10^{1}	3.1×10^{0}	1.1×10^{0}
900	2.7×10^{2}	3.6×10^{2}	9.3×10^{1}	3.7×10^{1}
1000	3.3×10^{3}	4.2×10^{3}	1.2×10^{3}	6.0×10^{2}
1100	2.5×10^{4}	3.1×10^{4}	1.2×10^{4}	5.9×10^{3}
1200	1.4×10^{5}	1.7×10^{5}	7.4×10^{4}	4.0×10^{4}
	TS41	TS42	TS43	TS44
600	1.6×10^{3}	8.5×10^{3}	8.4×10^{3}	8.5×10^{5}
700	2.0×10^{4}	2.2×10^{5}	1.5×10^{5}	9.3×10^{6}
800	1.3×10^{5}	2.6×10^{6}	1.4×10^{6}	5.8×10^{7}
900	5.4×10^{5}	1.8×10^{7}	7.6×10^{6}	2.4×10^{8}
1000	1.7×10^{6}	8.3×10^{7}	2.3×10^{7}	7.7×10^{8}
1100	4.5×10^{6}	2.9×10^{8}	7.1×10^{7}	2.0×10^{9}
1200	1.0×10^{7}	8.4×10^{8}	1.8×10^{8}	4.4×10^{9}
	TS45	TS46	TS47	TS48
600	4.4×10^{2}	1.0×10^{4}	2.1×10^{1}	1.4×10^{6}
700	1.1×10^{4}	2.9×10^{5}	9.6×10^{2}	1.5×10^{7}
800	1.1×10^{5}	2.8×10^{6}	1.7×10^{4}	9.0×10^{7}
900	7.3×10^{5}	1.9×10^{7}	1.6×10^{5}	3.7×10^{8}
1000	3.2×10^{6}	9.0×10^{7}	7.8×10^{5}	1.1×10^{9}
1100	1.1×10^{7}	3.2×10^{8}	3.4×10^{6}	2.9×10^{9}
1200	3.0×10^{7}	9.2×10^{8}	1.2×10^{7}	6.3×10^{9}

Table S7. Cartesian coordinates for the transition states involved in the NSO-HETs formation routes, x coordinate, y coordinate and z coordinate.

TS1

0	2		
C	-0.99429	0.21498	0.84085
C	-1.27295	-0.83063	-0.04817
C	-2.38411	-0.86117	-0.86116
C	-3.25268	0.21135	-0.76011
C	-3.00637	1.27056	0.11973
C	-1.88686	1.28791	0.92130
H	-2.56309	-1.68360	-1.53428
H	-4.13963	0.23046	-1.37401
H	-3.70820	2.08858	0.16457
H	-1.69870	2.11074	1.59288
O	-0.31800	-1.77788	0.01454
C	1.54921	0.22745	-1.34582
C	3.14652	-0.10728	0.30835
C	2.06362	1.42788	-0.98033
H	0.79294	0.04408	-2.08883
C	3.05955	1.21811	0.05348
H	3.79822	-0.59040	1.01633
H	1.79061	2.38698	-1.38810
H	3.63200	1.99871	0.52643
C	2.16079	-0.78028	-0.52014
H	2.24978	-1.81084	-0.82879
C	0.65388	-1.29992	0.85582
H	1.27761	-2.06262	1.28559
C	0.21976	-0.13213	1.46901
H	0.77603	0.42988	2.19495

TS2

0	2		
C	-0.56416	0.15882	-0.74289
C	-1.42603	0.72879	0.18261
C	-2.48139	0.06020	0.75796
C	-2.66438	-1.25379	0.35896
C	-1.82088	-1.85287	-0.57087
C	-0.75810	-1.15779	-1.12463
H	-3.12898	0.53973	1.47339
H	-3.48205	-1.82160	0.77521
H	-1.99590	-2.87710	-0.86070
H	-0.09677	-1.62822	-1.83401
O	-1.09606	2.02715	0.41616
C	0.43760	1.17458	-1.04195
H	0.94582	1.24332	-1.98901
C	2.11654	0.65682	-0.04338
H	2.62857	1.58906	-0.23144
C	1.65941	0.29105	1.28607
C	2.53312	-0.57483	-0.68177

H	1.29239	0.98430	2.02392
C	1.70509	-1.05558	1.38846
H	2.95949	-0.64700	-1.66815
C	2.25390	-1.59748	0.16080
H	1.38314	-1.64272	2.23208
H	2.41090	-2.64546	-0.03491
C	-0.06660	2.31669	-0.40655
H	0.30697	3.32094	-0.35257

TS3

0	2		
C	-0.77057	0.47577	0.62477
C	-1.00762	-0.83588	0.20895
C	-2.20149	-1.22885	-0.35417
C	-3.18476	-0.26068	-0.50092
C	-2.97691	1.05158	-0.08816
C	-1.77119	1.42780	0.47734
H	-2.35993	-2.25116	-0.65690
H	-4.13165	-0.53508	-0.93990
H	-3.76290	1.77998	-0.21196
H	-1.60686	2.44691	0.79347
O	0.02147	-1.66443	0.47459
C	1.58283	-0.47338	-1.42333
C	2.88058	0.48464	0.26249
C	1.37018	0.84824	-1.43615
H	1.18205	-1.19938	-2.10901
C	1.96506	1.41782	-0.23296
H	3.48980	0.61459	1.14270
H	0.77230	1.39718	-2.14402
H	1.98330	2.47477	-0.01994
C	2.26946	-0.81382	-0.14934
H	2.87415	-1.71292	-0.13186
C	1.10994	-0.86791	0.93255
H	1.49153	-1.32251	1.84242
C	0.57757	0.52805	1.11968
H	0.83794	1.10271	1.99374

TS4

0	2		
C	0.80745	0.49238	-0.54548
C	1.04633	-0.83919	-0.25227
C	2.23820	-1.29069	0.26460
C	3.22828	-0.34134	0.48003
C	3.01843	0.99765	0.18658
C	1.79874	1.42420	-0.32623
H	2.39132	-2.33526	0.48144
H	4.18069	-0.65597	0.87812
H	3.80747	1.71261	0.35918
H	1.63378	2.46886	-0.54475

O	-0.00960	-1.63581	-0.56646
C	-1.02194	-0.83969	-1.04606
H	-1.51532	-1.27266	-1.90139
C	-0.61435	0.60532	-0.98315
H	-0.74037	1.11941	-1.93379
C	-2.74707	-0.61188	0.26726
H	-3.41015	-1.44213	0.08495
C	-1.58592	1.31288	0.08815
H	-1.51013	2.39259	0.02382
C	-2.86233	0.64795	-0.30177
H	-3.41428	0.88544	-1.19700
C	-1.25273	0.67695	1.38895
H	-0.48432	1.02438	2.05794
C	-1.90133	-0.49249	1.45118
H	-1.77241	-1.26303	2.19256

TS5

0	2		
C	0.89118	0.58461	-0.36898
C	1.08412	-0.78564	-0.16798
C	2.30730	-1.30446	0.19697
C	3.35969	-0.41271	0.34947
C	3.19024	0.95059	0.15245
C	1.94766	1.45772	-0.19923
H	2.43314	-2.36370	0.35229
H	4.33229	-0.79155	0.62424
H	4.02857	1.61794	0.27583
H	1.80708	2.51805	-0.34485
O	-0.03554	-1.53113	-0.33874
H	-1.50340	1.19404	-1.47672
C	-0.54643	0.74244	-0.60893
C	-2.43275	-0.63901	-0.44493
H	-3.11465	-1.30438	-0.96066
C	-1.58299	1.26832	0.40632
H	-1.47873	2.32607	0.61752
C	-2.63554	0.86572	-0.61285
H	-3.62302	1.30971	-0.67464
C	-1.82161	0.33770	1.55113
H	-1.53356	0.51183	2.57486
C	-2.36579	-0.77159	1.05578
H	-2.59791	-1.67765	1.59071
C	-0.98119	-0.66573	-0.95025
H	-0.94879	-0.85905	-2.02256

TS6

0	2		
C	0.75374	0.42411	-0.47940
C	1.12157	-0.87357	-0.14189
C	2.38344	-1.19702	0.30164

C	3.30249	-0.16214	0.40919
C	2.96119	1.14021	0.07892
C	1.68321	1.43733	-0.37779
H	2.63603	-2.21274	0.55910
H	4.29996	-0.37859	0.75984
H	3.69404	1.92616	0.17309
H	1.42244	2.45016	-0.64660
O	0.11169	-1.78427	-0.29017
H	-2.13205	-1.03193	-1.34253
C	-2.10700	-0.85690	0.54560
H	-2.54951	-1.79155	0.86878
C	-1.88478	1.11348	-0.52245
H	-2.15030	1.97979	-1.11676
C	-2.85576	-0.06736	-0.51404
H	-3.93071	0.06632	-0.50987
C	-1.69234	1.34499	0.95699
H	-1.40468	2.29002	1.38869
C	-1.76555	0.16893	1.57715
H	-1.55361	-0.02651	2.61544
C	-1.03569	-1.04194	-0.53539
C	-0.63452	0.34013	-1.01847
H	-0.60506	0.38164	-2.10819

TS7

0	2		
C	0.96266	-0.53179	0.28620
C	1.23850	0.81648	-0.01603
C	2.51119	1.26052	-0.28858
C	3.53715	0.32519	-0.25635
C	3.28806	-1.01268	0.02858
C	2.00249	-1.45314	0.28871
C	-0.46180	-0.59331	0.36903
C	-0.88643	0.81409	0.53926
H	2.69320	2.29666	-0.52356
H	4.54745	0.64564	-0.45848
H	4.10695	-1.71470	0.04369
H	1.80997	-2.49386	0.49880
C	-2.93312	-0.10357	1.16599
H	-3.89333	-0.53841	0.93614
C	-2.37045	0.89563	0.17301
C	-1.47798	-1.46413	-0.01423
H	-2.76889	1.90234	0.29757
C	-2.65599	0.28207	-1.15770
H	-1.33540	-2.53304	0.07923
C	-2.27704	-0.99229	-1.16498
H	-3.11454	0.80510	-1.97918
H	-2.41924	-1.67169	-1.99239
H	-0.84516	1.03116	1.61472
H	-2.69347	0.00632	2.21708

O	0.13113	1.60075	-0.05639

TS8

0	2		
C	-0.86007	-0.50393	-0.24727
C	-1.28996	0.81839	-0.15505
C	-2.59405	1.16578	0.10043
C	-3.49611	0.12694	0.28988
C	-3.09413	-1.19744	0.21555
C	-1.77245	-1.52095	-0.06877
C	0.56219	-0.42368	-0.70812
C	0.85886	0.97254	-0.34001
H	-2.89185	2.19960	0.16421
H	-4.52753	0.35876	0.50621
H	-3.81514	-1.98411	0.37342
H	-1.46716	-2.55344	-0.14605
C	2.90046	-0.37537	-1.06738
H	3.92827	-0.50955	-0.76426
C	2.07576	1.23349	0.26609
C	1.85067	-1.18974	-0.33097
H	2.37996	2.26435	0.39390
C	2.41677	0.28638	1.35647
H	1.81273	-2.23384	-0.64177
C	2.20090	-1.00156	1.11110
H	2.70713	0.69730	2.31204
H	2.26128	-1.79858	1.83232
H	0.53297	-0.47583	-1.80570
H	2.73958	-0.14605	-2.11443
O	-0.27667	1.72954	-0.33019

TS9

0	2		
C	1.04281	0.58574	0.12044
C	1.23891	-0.76685	-0.15172
C	2.48963	-1.33200	-0.26165
C	3.57597	-0.48746	-0.09863
C	3.40832	0.86834	0.16549
C	2.14217	1.41594	0.27366
H	2.60898	-2.38268	-0.46936
H	4.57327	-0.89196	-0.17682
H	4.27576	1.49751	0.28832
H	2.01271	2.46775	0.47763
O	0.07296	-1.44003	-0.30970
C	-0.38790	0.78422	0.10546
C	-2.34317	-0.84789	-0.37769
H	-2.42823	-1.42153	-1.30099
C	-2.61308	1.63322	-0.19398
H	-3.26078	2.49587	-0.24336
C	-3.13026	0.42007	-0.44696

H	-4.17408	0.32579	-0.70927
C	-1.21062	1.85555	0.05628
H	-0.83315	2.86332	0.12708
C	-0.93602	-0.55746	0.06581
H	-1.33449	-0.96680	1.24848
C	-2.64067	-1.64306	0.88427
H	-2.37768	-2.69231	0.87079
H	-3.46262	-1.34492	1.52054

TS10

0	2		
C	1.04181	0.59940	0.04876
C	1.25375	-0.74214	-0.25874
C	2.50063	-1.32675	-0.28310
C	3.57354	-0.50953	0.02445
C	3.39321	0.83659	0.33858
C	2.13264	1.40259	0.35336
H	2.62555	-2.36854	-0.52899
H	4.56995	-0.92311	0.02054
H	4.25307	1.44457	0.57255
H	2.00072	2.44590	0.59530
O	0.08770	-1.38061	-0.53108
C	-0.38486	0.78822	-0.04965
C	-2.25349	-0.72700	-0.54668
H	-2.57313	-1.62781	-1.04501
C	-2.60795	1.64869	-0.12262
H	-3.29722	2.47495	-0.04109
C	-3.09260	0.40185	-0.48709
H	-4.14575	0.27684	-0.68469
C	-1.25823	1.86328	0.11196
H	-0.89358	2.84017	0.38718
C	-0.88873	-0.44916	-0.41123
C	-2.62242	-1.77203	1.34777
H	-3.66036	-2.05155	1.24811
H	-1.89884	-2.57082	1.28311
H	-2.40592	-0.97973	2.04695

TS11

0	2		
C	-1.03897	0.61891	0.09318
C	-1.19871	-0.76466	0.15247
C	-2.42450	-1.37442	0.00741
C	-3.52224	-0.55161	-0.20074
C	-3.38863	0.83023	-0.26475
C	-2.14586	1.42455	-0.12500
H	-2.51808	-2.44706	0.05512
H	-4.49925	-0.99601	-0.31209
H	-4.26115	1.44388	-0.42454
H	-2.03962	2.49729	-0.17877

O	-0.03312	-1.42729	0.33773
C	0.37270	0.86734	0.22752
C	2.29386	-0.70684	0.01759
C	2.59177	1.71677	-0.06239
H	3.24258	2.56870	-0.17978
C	3.11257	0.46330	-0.13946
H	4.15741	0.32414	-0.37587
C	1.17237	1.92218	-0.00169
H	0.76437	2.89313	-0.23851
C	0.95337	-0.45095	0.65128
H	1.05476	-0.44952	1.74410
C	2.57402	-1.99081	-0.63647
H	3.45449	-2.10372	-1.24407
H	2.91992	-1.53767	0.63961
H	1.87899	-2.79877	-0.50065

TS12

0	2		
C	-1.07848	0.60180	0.01800
C	-1.20188	-0.78632	0.00233
C	-2.41875	-1.43219	-0.03241
C	-3.54691	-0.63147	-0.05920
C	-3.45274	0.75954	-0.04850
C	-2.22363	1.38729	-0.01029
H	-2.47959	-2.50806	-0.03945
H	-4.52056	-1.09540	-0.08709
H	-4.35476	1.35068	-0.06853
H	-2.15442	2.46401	-0.00050
O	0.00585	-1.40170	0.01742
C	0.33316	0.86107	0.04421
C	2.31104	-0.61626	-0.04705
C	2.50442	1.81039	-0.04321
H	3.15307	2.67165	-0.07969
C	3.07260	0.53270	-0.08997
H	4.14519	0.43896	-0.17472
C	1.13528	1.98883	0.00438
H	0.70541	2.97792	-0.01842
C	0.93633	-0.40826	0.14027
H	1.00872	-0.46204	1.87715
C	2.86836	-1.99353	-0.08919
H	2.69784	-2.50286	0.85902
H	2.38336	-2.58751	-0.86072
H	3.93616	-1.97516	-0.28267

TS13

0	2		
C	-0.83380	0.40022	-0.04826
C	-1.32355	-0.88761	0.13838
C	-2.66298	-1.18962	0.11326

C	-3.53735	-0.13609	-0.12107
C	-3.07422	1.15737	-0.31888
C	-1.71568	1.43870	-0.27596
H	-3.01048	-2.19939	0.25806
H	-4.59785	-0.33130	-0.15670
H	-3.77898	1.95242	-0.50503
H	-1.35667	2.44682	-0.42046
O	-0.32461	-1.79476	0.32719
C	0.85153	-1.12666	0.15565
C	2.99241	0.52351	-0.54266
H	3.83818	1.12340	-0.84607
C	3.14597	-0.79281	-0.34967
H	4.12243	-1.23120	-0.49232
C	1.69806	1.24555	-0.34127
C	2.05175	-1.68635	-0.03234
H	2.18885	-2.75428	-0.02205
H	1.43371	1.83105	-1.22313
C	0.61533	0.30590	0.15143
C	1.68508	2.03827	0.95376
H	0.96388	2.84113	1.03691
H	2.59138	2.12300	1.53679
H	0.84707	0.81410	1.32230

TS14

0	2		
C	-0.84385	0.35927	-0.25620
C	-1.33425	-0.88960	0.12523
C	-2.67405	-1.15582	0.29649
C	-3.54827	-0.10607	0.07373
C	-3.08807	1.15434	-0.30446
C	-1.73868	1.39955	-0.47356
H	-3.01613	-2.13446	0.59103
H	-4.60779	-0.26791	0.19674
H	-3.79949	1.94863	-0.46782
H	-1.38926	2.37719	-0.76875
O	-0.33079	-1.78270	0.30538
C	0.82246	-1.11671	0.04282
C	2.94494	0.47725	-0.61033
H	3.79186	1.08438	-0.88923
C	3.14226	-0.84386	-0.24832
H	4.14355	-1.24633	-0.25331
C	1.66703	1.06365	-0.56494
C	2.08360	-1.67535	0.10030
H	2.22929	-2.70643	0.37581
H	1.49868	2.01582	-1.04332
C	0.58154	0.20175	-0.31616
C	1.74370	2.18792	1.34434
H	0.74147	2.58818	1.38266
H	1.96264	1.37211	2.01463

H	2.54138	2.89256	1.16487

TS15

0	2		
C	-0.82376	0.35992	0.22665
C	-1.28325	-0.92413	-0.02575
C	-2.60460	-1.22313	-0.26101
C	-3.50249	-0.16695	-0.23266
C	-3.07901	1.12446	0.03950
C	-1.73833	1.39244	0.28541
H	-2.91677	-2.23605	-0.45549
H	-4.54818	-0.35802	-0.41776
H	-3.79752	1.92812	0.07091
H	-1.43456	2.39655	0.53457
O	-0.29192	-1.85538	-0.00378
C	0.88437	-1.18810	0.15286
C	3.01354	0.53944	-0.10356
H	3.85845	1.20296	-0.21777
C	3.20897	-0.80042	-0.01886
H	4.21175	-1.19665	-0.03717
C	1.69125	1.10815	-0.11061
C	2.09562	-1.71224	-0.02844
H	2.22868	-2.75739	-0.25448
C	0.64451	0.24410	0.52796
H	0.78200	0.35385	1.61846
C	1.45990	2.54715	-0.27709
H	1.37004	1.54700	-1.22293
H	0.49280	2.97495	-0.09735
H	2.28843	3.18368	-0.53717

TS16

0	2		
C	-0.80533	0.36337	0.05317
C	-1.30994	-0.93189	0.00628
C	-2.65306	-1.22180	-0.06899
C	-3.51905	-0.14275	-0.10220
C	-3.04523	1.16461	-0.05313
C	-1.68971	1.43045	0.03315
H	-3.00215	-2.24076	-0.10271
H	-4.58124	-0.32051	-0.16600
H	-3.74715	1.98325	-0.07705
H	-1.33784	2.44776	0.09009
O	-0.32117	-1.86296	0.03632
C	0.84911	-1.18428	0.05467
C	2.98655	0.49400	-0.11703
H	3.85191	1.13176	-0.21976
C	3.16742	-0.89078	-0.09033
H	4.16679	-1.29112	-0.15861
C	1.73946	1.07476	-0.03308

C	2.09084	-1.76016	-0.02844
H	2.21063	-2.82988	-0.07335
C	0.64115	0.20575	0.18023
H	0.63617	0.32805	1.87369
C	1.53745	2.54799	-0.05117
H	0.87476	2.84484	-0.86292
H	1.07985	2.88196	0.88117
H	2.47970	3.07272	-0.17403

TS17

0	2		
C	-0.97282	0.22238	0.82124
C	-1.25148	-0.82322	-0.06777
C	-2.36264	-0.85377	-0.88076
C	-3.23121	0.21875	-0.77971
C	-2.98490	1.27796	0.10013
C	-1.86539	1.29532	0.90170
H	-2.54162	-1.67620	-1.55388
H	-4.11816	0.23786	-1.39361
H	-3.68673	2.09598	0.14496
H	-1.67723	2.11814	1.57328
C	1.52774	0.22005	-1.32621
C	3.12505	-0.11468	0.32795
C	2.04215	1.42047	-0.96072
H	0.77147	0.03667	-2.06923
C	3.03808	1.21071	0.07309
H	3.77675	-0.59781	1.03594
H	1.76914	2.37957	-1.36850
H	3.61053	1.99131	0.54603
C	2.07287	-0.81059	-0.43985
H	2.22831	-1.81824	-0.80918
C	0.74180	-1.26960	0.77554
H	1.29908	-2.05522	1.26599
C	0.24123	-0.12472	1.44941
H	0.79750	0.43728	2.17534
S	-0.29653	-1.77048	-0.00507

TS18

0	2		
C	-0.56416	0.15882	-0.74289
C	-1.42603	0.72879	0.18261
C	-2.48139	0.06020	0.75796
C	-2.66438	-1.25379	0.35896
C	-1.82088	-1.85287	-0.57087
C	-0.75810	-1.15779	-1.12463
H	-3.12898	0.53973	1.47339
H	-3.48205	-1.82160	0.77521
H	-1.99590	-2.87710	-0.86070
H	-0.09677	-1.62822	-1.83401

C	0.49198	1.15781	-1.00961
H	0.94582	1.24332	-1.98901
C	2.06216	0.67358	-0.07572
H	2.62857	1.58906	-0.23144
C	1.65941	0.29105	1.28607
C	2.53312	-0.57483	-0.68177
H	1.29239	0.98430	2.02392
C	1.70509	-1.05558	1.38846
H	2.95949	-0.64700	-1.66815
C	2.25390	-1.59748	0.16080
H	1.38314	-1.64272	2.23208
H	2.41090	-2.64546	-0.03491
C	-0.06660	2.31669	-0.40655
H	0.30697	3.32094	-0.35257
S	-1.09606	2.02715	0.41616

TS19

0	2		
C	-0.78279	-0.43994	-0.63123
C	-1.14927	0.77574	-0.04007
C	-2.39361	0.94346	0.53908
C	-3.29157	-0.11151	0.51553
C	-2.95199	-1.31698	-0.08607
C	-1.70466	-1.48258	-0.65693
C	0.56813	-0.45437	-1.13314
C	1.29821	0.82218	-0.80749
H	-2.66652	1.88434	0.99119
H	-4.26557	0.01011	0.96391
H	-3.66371	-2.12763	-0.10323
H	-1.43557	-2.42297	-1.11508
S	0.06277	2.02485	-0.22784
C	2.85736	-0.82260	-0.30926
H	3.44965	-0.90884	-1.20638
C	2.41476	0.47724	0.27592
C	1.83038	-1.68789	0.06856
H	3.13295	1.28373	0.36983
C	1.68844	0.06117	1.50477
H	1.70905	-2.69856	-0.28749
C	1.30702	-1.21130	1.34606
H	1.38550	0.73386	2.28777
H	0.64480	-1.77067	1.98505
H	1.79108	1.24268	-1.67869
H	0.77162	-0.93949	-2.07578

TS20

0	2		
C	-0.83247	-0.47280	-0.55030
C	-1.18585	0.78304	-0.07176
C	-2.43803	1.02275	0.46425

C	-3.35429	-0.01614	0.50470
C	-3.02048	-1.27005	0.01889
C	-1.75713	-1.49896	-0.50534
C	0.58956	-0.57700	-0.99679
C	1.19915	0.79882	-0.94912
H	-2.70136	2.00073	0.83606
H	-4.33711	0.15851	0.91471
H	-3.74316	-2.07042	0.05076
H	-1.49358	-2.47977	-0.87455
S	0.08977	1.97117	-0.26769
C	2.82082	-0.96902	-0.37206
H	3.32929	-1.17698	-1.29992
C	2.85726	0.24021	0.31545
C	1.47486	-1.50346	-0.02504
H	3.61668	0.99711	0.20162
C	2.03196	0.09254	1.51084
H	1.26211	-2.55310	-0.19645
C	1.24060	-0.97380	1.34370
H	2.01135	0.78887	2.33185
H	0.44528	-1.29545	1.99391
H	1.75110	1.15810	-1.80337
H	0.66722	-1.00314	-1.99606

TS21

0	2		
C	-0.93464	-0.59552	-0.31626
C	-1.27656	0.73572	-0.05025
C	-2.57637	1.07959	0.27303
C	-3.54064	0.08448	0.32534
C	-3.21312	-1.23637	0.06334
C	-1.90779	-1.57760	-0.25188
C	0.50283	-0.74477	-0.55048
C	1.10861	0.59296	-0.91802
H	-2.83872	2.10595	0.47791
H	-4.55882	0.34687	0.56888
H	-3.97498	-1.99903	0.10299
H	-1.64542	-2.60482	-0.45660
S	0.07862	1.86061	-0.12154
C	2.54488	-1.19297	-0.67757
H	3.45377	-1.77696	-0.77695
C	2.57920	0.32493	-0.51177
C	1.50799	-1.42441	0.40768
H	3.31334	0.87970	-1.08377
C	2.65881	0.45218	0.98851
H	1.24637	-2.45224	0.63130
C	1.97327	-0.55270	1.52819
H	3.07578	1.30310	1.50204
H	1.73226	-0.68250	2.57040
H	1.04837	0.76940	-1.98905

H	1.33121	-1.33237	-1.46165

TS22

0	2		
C	-0.74952	-0.43719	-0.48460
C	-1.26475	0.78268	-0.05188
C	-2.57165	0.89178	0.38691
C	-3.36868	-0.24219	0.39884
C	-2.86689	-1.46223	-0.02359
C	-1.55840	-1.55707	-0.47280
C	0.64466	-0.31303	-1.00269
C	1.22304	0.96629	-0.41686
H	-2.96254	1.83881	0.72535
H	-4.38756	-0.17096	0.74751
H	-3.49476	-2.33942	-0.00403
H	-1.16828	-2.50651	-0.81027
S	-0.09691	2.11241	-0.11357
C	2.90624	-0.23446	-0.50963
H	3.95752	-0.49767	-0.54499
C	2.26982	0.53629	0.63161
C	1.79764	-1.27753	-0.61406
H	2.82866	1.37067	1.03793
C	1.80605	-0.53268	1.56557
H	1.94895	-2.10731	-1.29471
C	1.58579	-1.62695	0.83943
H	1.62386	-0.41064	2.62049
H	1.18237	-2.56419	1.18720
H	2.29969	0.85573	-1.23410
H	0.60254	-0.22932	-2.09067

TS23

0	2		
C	-0.96932	-0.59957	-0.15924
C	-1.40907	0.72669	0.02505
C	-2.75236	1.01475	0.15949
C	-3.67082	-0.02350	0.11353
C	-3.24959	-1.33530	-0.05715
C	-1.90421	-1.62835	-0.18336
C	0.46425	-0.63818	-0.18615
C	1.01173	0.67548	-0.59726
H	-3.08352	2.03205	0.29967
H	-4.72323	0.19395	0.21035
H	-3.97667	-2.13157	-0.09353
H	-1.57658	-2.64880	-0.31367
S	-0.10031	1.90798	0.13955
C	2.85176	-0.62992	-1.26740
H	3.78551	-1.13602	-1.07416
C	2.53346	0.59289	-0.43214
C	1.41599	-1.53986	0.26527

H	3.03116	1.49666	-0.78291
C	2.90672	0.19400	0.96041
H	1.15238	-2.58537	0.36331
C	2.37957	-0.99079	1.24539
H	3.51311	0.78879	1.62188
H	2.51975	-1.52298	2.17436
H	0.83773	0.75708	-1.67408
H	2.47671	-0.68142	-2.28266

TS24

0	2		
C	-0.82514	-0.52662	-0.27900
C	-1.43481	0.71154	-0.06060
C	-2.78270	0.80822	0.22109
C	-3.52773	-0.35851	0.31006
C	-2.93267	-1.59309	0.11377
C	-1.58151	-1.67780	-0.19265
C	0.59420	-0.34692	-0.70749
C	1.01306	0.95976	-0.13898
H	-3.24620	1.76804	0.38878
H	-4.57909	-0.29969	0.54596
H	-3.52154	-2.49338	0.19575
H	-1.12146	-2.63990	-0.36321
S	-0.31509	2.08531	-0.16789
C	2.93209	-0.48895	-1.11051
H	3.93908	-0.75981	-0.83063
C	2.25023	0.97309	0.47820
C	1.79822	-1.28278	-0.49725
H	2.71305	1.91861	0.72775
C	2.48918	-0.16328	1.40149
H	1.64341	-2.25268	-0.97134
C	2.15815	-1.36815	0.95369
H	2.80891	0.05976	2.40858
H	2.14158	-2.27390	1.53575
H	0.55685	-0.18861	-1.79527
H	2.80347	-0.08242	-2.10682

TS25

0	2		
C	-1.04102	0.59693	0.08851
C	-1.42515	-0.72772	-0.13645
C	-2.75871	-1.09595	-0.15404
C	-3.71922	-0.12108	0.04888
C	-3.35271	1.20085	0.26986
C	-2.01923	1.56136	0.28893
C	0.39763	0.76959	0.02537
C	1.06473	-0.52177	0.09924
H	-3.04518	-2.12232	-0.32226
H	-4.76301	-0.39457	0.04100

H	-4.11343	1.94801	0.43356
H	-1.73407	2.58730	0.46750
S	-0.06686	-1.79842	-0.41162
C	2.55205	-0.65063	-0.19183
C	1.11985	1.90324	-0.12096
C	3.19687	0.66888	-0.44815
H	0.63032	2.86493	-0.14298
C	2.54007	1.83248	-0.35119
C	2.83110	-1.17333	1.20709
H	3.45693	-0.58956	1.86758
H	1.38038	-0.78494	1.32986
H	3.07443	2.75690	-0.51127
H	4.24762	0.65688	-0.69879
H	2.79965	-1.35961	-0.98131
H	2.81857	-2.24552	1.35155

TS26

0	2		
C	-1.04766	0.59775	0.01608
C	-1.43283	-0.73601	-0.14841
C	-2.76315	-1.12063	-0.07461
C	-3.71514	-0.15316	0.17324
C	-3.34815	1.18023	0.34123
C	-2.02477	1.55724	0.26251
C	0.38191	0.76542	-0.10840
C	1.02148	-0.44287	-0.36509
H	-3.04816	-2.15281	-0.20563
H	-4.75468	-0.43494	0.23722
H	-4.10690	1.92261	0.53342
H	-1.74579	2.59213	0.39111
S	-0.07672	-1.78297	-0.44925
C	2.42435	-0.53738	-0.45500
C	1.13760	1.93704	-0.02768
C	3.13596	0.67338	-0.49343
H	0.65588	2.88263	0.16772
C	2.50473	1.87824	-0.23935
C	2.92199	-1.29824	1.55103
H	2.53716	-0.48398	2.14464
H	2.35597	-2.21758	1.57142
H	3.08481	2.78805	-0.22232
H	4.19975	0.64976	-0.67177
H	2.87501	-1.43262	-0.85378
H	3.99648	-1.39170	1.50319

TS27

0	2		
C	-1.09479	0.59738	0.08379
C	-1.38117	-0.76832	-0.00097
C	-2.68362	-1.22156	-0.11142

C	-3.71229	-0.29573	-0.13781
C	-3.44326	1.06448	-0.05042
C	-2.14122	1.51138	0.05978
C	0.32310	0.86430	0.15283
C	1.09170	-0.37384	0.50622
H	-2.89480	-2.27762	-0.17461
H	-4.73238	-0.63779	-0.21989
H	-4.25568	1.77409	-0.06144
H	-1.93409	2.56806	0.13722
S	0.04686	-1.78410	-0.00169
C	2.47701	-0.38124	-0.05388
C	0.97772	2.02541	-0.04308
C	3.12103	0.89574	-0.11947
H	0.42856	2.93583	-0.23262
C	2.40530	2.05153	-0.08272
H	1.17273	-0.44153	1.60017
C	3.20531	-1.65061	-0.08345
H	2.69427	-2.57173	0.13473
H	2.91419	2.99830	-0.16957
H	4.19253	0.92446	-0.25453
H	4.25460	-1.65893	-0.32401
H	2.54136	-0.98117	-1.12476

TS28

0	2		
C	-1.09817	0.59165	0.01575
C	-1.37922	-0.77878	-0.01672
C	-2.68506	-1.24536	-0.04151
C	-3.71477	-0.32639	-0.04245
C	-3.45049	1.04121	-0.01345
C	-2.15226	1.50086	0.01610
C	0.31627	0.85528	0.03445
C	1.06905	-0.33632	0.14822
H	-2.89269	-2.30376	-0.06083
H	-4.73607	-0.67381	-0.06257
H	-4.26910	1.74370	-0.01062
H	-1.95096	2.56101	0.04311
S	0.05636	-1.76279	-0.03510
C	2.47046	-0.34003	-0.02962
C	0.98440	2.06967	-0.01245
C	3.08921	0.88735	-0.08542
H	0.43106	2.99568	-0.04486
C	2.36261	2.08041	-0.05300
H	1.13481	-0.36827	1.87865
C	3.21069	-1.62963	-0.04834
H	3.07199	-2.16260	0.89335
H	2.84360	-2.27921	-0.84259
H	2.88960	3.02068	-0.09663
H	4.16552	0.92552	-0.16680

H	4.27425	-1.47380	-0.19809

TS29

0	2		
C	-0.80498	0.46543	-0.03895
C	-1.45555	-0.76461	0.07428
C	-2.83084	-0.85867	-0.01521
C	-3.56372	0.29758	-0.23169
C	-2.92938	1.52450	-0.35498
C	-1.55071	1.61247	-0.25277
C	0.64197	0.34302	0.17353
C	1.03041	-1.05526	0.05607
H	-3.32604	-1.81352	0.06783
H	-4.63791	0.23767	-0.31345
H	-3.51149	2.41552	-0.53099
H	-1.05951	2.57052	-0.34470
S	-0.34858	-2.10249	0.31527
C	2.28973	-1.43057	-0.23235
C	1.64096	1.42969	-0.19830
C	3.27211	-0.41607	-0.53758
C	2.97668	0.88830	-0.58105
H	3.72626	1.60487	-0.88350
H	4.26821	-0.74335	-0.79558
H	2.56078	-2.47165	-0.30480
H	1.27381	2.12318	-0.95558
C	1.62221	2.00430	1.20520
H	2.52524	1.96634	1.79761
H	0.90955	2.78792	1.42583
H	0.81499	0.72161	1.39584

TS30

0	2		
C	-0.80698	0.41872	-0.23579
C	-1.45717	-0.78564	0.05971
C	-2.83795	-0.85638	0.15772
C	-3.57280	0.29580	-0.03798
C	-2.94134	1.50403	-0.32504
C	-1.56808	1.57025	-0.42375
C	0.61973	0.25830	-0.28911
C	1.01265	-1.05036	-0.02098
H	-3.32901	-1.78990	0.38403
H	-4.64860	0.25856	0.03531
H	-3.53314	2.39417	-0.47094
H	-1.08146	2.50867	-0.64589
S	-0.34194	-2.10125	0.27950
C	2.35017	-1.42910	-0.03076
C	1.60279	1.24921	-0.50082
C	3.30090	-0.46980	-0.34600
C	2.93853	0.83773	-0.61675

H	3.69453	1.56197	-0.87658
H	4.34031	-0.75581	-0.39737
H	2.64019	-2.44742	0.17331
H	1.30751	2.20348	-0.90959
C	1.58357	2.24266	1.48363
H	1.92012	1.41655	2.08945
H	2.27909	3.05651	1.34594
H	0.53737	2.50063	1.55577

TS31

0	2		
C	-0.80656	0.40049	-0.30302
C	-1.38760	-0.80457	0.07787
C	-2.75119	-0.91244	0.28664
C	-3.54467	0.20726	0.10537
C	-2.98416	1.41084	-0.29041
C	-1.61746	1.50377	-0.50111
C	0.66456	0.30566	-0.55759
C	1.10104	-1.07414	-0.13441
H	-3.18973	-1.85223	0.58384
H	-4.60883	0.13628	0.26924
H	-3.61113	2.27570	-0.43993
H	-1.18723	2.43967	-0.82843
S	-0.24600	-2.12565	0.22950
C	2.40375	-1.37915	-0.08649
C	1.60281	1.32200	0.05765
C	3.36789	-0.31665	-0.23312
C	2.99555	0.97668	-0.08718
H	3.73956	1.75383	0.01407
H	4.41239	-0.56831	-0.33094
H	2.74493	-2.37801	0.13709
H	1.41473	2.48366	-0.31961
C	1.17943	2.35857	1.01270
H	0.13353	2.54055	1.17644
H	1.91881	2.84130	1.62781
H	0.82868	0.34794	-1.64307

TS32

0	2		
C	0.78016	0.43279	0.04109
C	1.43057	-0.80416	-0.00929
C	2.81050	-0.90938	-0.07532
C	3.56197	0.24707	-0.09492
C	2.93823	1.48733	-0.03960
C	1.56309	1.58461	0.03501
C	-0.67514	0.27294	0.15010
C	-1.03333	-1.09557	0.02506
H	3.28593	-1.87699	-0.11340
H	4.63764	0.18588	-0.15106

H	3.53280	2.38747	-0.04903
H	1.11084	2.55817	0.09818
S	0.33669	-2.15396	0.02572
C	-2.34854	-1.51087	-0.04426
C	-1.70142	1.24424	-0.02833
C	-3.33603	-0.54905	-0.09079
C	-3.00307	0.80467	-0.10552
H	-3.79130	1.53819	-0.18754
H	-4.37148	-0.84580	-0.14969
H	-2.59335	-2.56051	-0.08633
C	-1.41052	2.70488	-0.02498
H	-0.77398	2.99189	-0.85990
H	-2.33303	3.27250	-0.09827
H	-0.64356	0.35461	1.86084
H	-0.90629	3.00091	0.89517

TS33

0	2		
C	0.56624	-0.15575	-0.73108
C	1.44908	-0.72829	0.19097
C	2.50077	-0.02174	0.74794
C	2.66332	1.29108	0.34551
C	1.80278	1.87715	-0.57841
C	0.74513	1.16224	-1.11446
C	-0.44687	-1.15526	-1.04985
C	0.03229	-2.34125	-0.44881
H	3.17223	-0.47413	1.46155
H	3.47733	1.87018	0.75385
H	1.95984	2.90268	-0.87371
H	0.06912	1.62117	-1.81789
H	-0.93775	-1.18746	-2.00854
H	-0.39135	-3.32756	-0.46515
N	1.07569	-2.03990	0.37135
H	1.55381	-2.69572	0.95442
C	-2.10480	-0.64976	-0.06364
C	-1.66839	-0.31004	1.27923
C	-1.71691	1.03593	1.41039
C	-2.25399	1.60193	0.19189
C	-2.52613	0.59577	-0.67362
H	-1.40357	1.60492	2.26977
H	-2.40626	2.65400	0.01454
H	-2.93950	0.68950	-1.66401
H	-2.62640	-1.57318	-0.26969
H	-1.31228	-1.01721	2.00915

TS34

0	2		
C	-1.34662	-0.93859	0.01866
C	-0.92176	0.19412	-0.71245

C	-1.68731	1.34763	-0.77539
C	-2.89672	1.34992	-0.10967
C	-3.34395	0.23138	0.60581
C	-2.58225	-0.91019	0.67698
C	-0.32901	-1.89999	-0.09491
C	0.73936	-1.30318	-0.80100
H	-1.35232	2.21315	-1.32547
H	-3.51413	2.23438	-0.14279
H	-4.29770	0.27376	1.10814
H	-2.92415	-1.76997	1.23261
H	-0.31525	-2.89025	0.32296
H	1.44980	-1.86072	-1.38735
N	0.27880	-0.09802	-1.30221
H	0.93867	0.61916	-1.55211
C	3.11986	-0.15300	-0.24813
C	2.97668	1.18815	-0.10005
C	1.96631	1.43424	0.90901
C	1.50353	0.24283	1.36492
C	2.14603	-0.80290	0.60962
H	1.64384	2.40894	1.23583
H	0.73443	0.08876	2.10165
H	2.28646	-1.80226	0.99202
H	3.53243	1.95037	-0.62134
H	3.80154	-0.66161	-0.90901

TS35

0	2		
C	-0.81655	-0.45746	-0.54562
C	-1.07988	0.87362	-0.22261
C	-2.29830	1.26614	0.29645
C	-3.26843	0.28847	0.47635
C	-3.02509	-1.03603	0.15353
C	-1.78608	-1.41359	-0.35823
H	-2.49708	2.29643	0.54940
H	-4.23129	0.57189	0.87343
H	-3.79631	-1.77574	0.29964
H	-1.59169	-2.44782	-0.60287
C	1.06835	0.87411	-1.02497
H	1.60317	1.26831	-1.87600
C	0.61042	-0.56306	-0.98412
H	0.72824	-1.06298	-1.94320
C	2.76606	0.57349	0.25208
H	3.46088	1.38138	0.08312
C	1.56542	-1.32059	0.06703
H	1.44920	-2.39575	-0.01510
C	2.86408	-0.69720	-0.31758
H	3.38871	-0.93083	-1.23081
C	1.26848	-0.70003	1.38516
H	0.50280	-1.03963	2.06138

C	1.95166	0.44893	1.45701
H	1.85777	1.20554	2.21850
N	0.02495	1.63876	-0.53075
H	0.03022	2.63717	-0.50905

TS36

0	2		
C	-0.76725	-0.42343	-0.65563
C	-1.02873	0.87960	-0.20632
C	-2.23393	1.20130	0.39186
C	-3.18767	0.20298	0.52111
C	-2.95392	-1.08609	0.05460
C	-1.74275	-1.40325	-0.53609
H	-2.43592	2.20492	0.73431
H	-4.13388	0.43620	0.98532
H	-3.71782	-1.84052	0.15896
H	-1.55028	-2.40708	-0.88504
C	1.15871	0.91375	-0.91524
H	1.63893	1.32294	-1.80132
C	0.58836	-0.46367	-1.14299
H	0.84921	-1.01704	-2.03076
C	2.28193	0.77717	0.21853
H	2.91247	1.65943	0.26010
C	1.92699	-1.44733	0.18166
H	1.92911	-2.49276	-0.08274
C	2.87956	-0.51194	-0.23915
H	3.50061	-0.60723	-1.11575
C	1.32240	-0.92679	1.40173
H	0.69843	-1.49624	2.06979
C	1.56440	0.38904	1.46037
H	1.17145	1.07949	2.18762
N	0.01493	1.71991	-0.53513
H	0.18451	2.51045	0.05873

TS37

0	2		
C	-0.90323	-0.54572	-0.34643
C	-1.13165	0.82021	-0.11824
C	-2.39164	1.27673	0.22657
C	-3.41888	0.35054	0.33560
C	-3.20059	-1.00224	0.12094
C	-1.93221	-1.45539	-0.21483
H	-2.57311	2.32484	0.41130
H	-4.40892	0.69392	0.59520
H	-4.01732	-1.70050	0.21433
H	-1.75257	-2.50630	-0.38567
C	1.00175	0.72361	-0.91591
H	0.97540	0.90865	-1.99317
C	0.53432	-0.68718	-0.60118

H	1.44651	-1.15935	-1.49881
C	2.47851	0.60934	-0.45781
H	3.17382	1.25615	-0.98108
C	1.57693	-1.27595	0.37435
H	1.43129	-2.33339	0.56243
C	2.61744	-0.89554	-0.66484
H	3.58610	-1.37621	-0.75108
C	1.89024	-0.38682	1.53292
H	1.62139	-0.57067	2.56022
C	2.47042	0.70781	1.04566
H	2.75692	1.58891	1.59638
N	0.03963	1.56215	-0.23700
H	-0.06538	2.50248	-0.57186

TS38

0	2		
C	-0.76310	-0.39336	-0.48483
C	-1.15259	0.90396	-0.14335
C	-2.43118	1.17531	0.30458
C	-3.32318	0.11710	0.41248
C	-2.95236	-1.17273	0.07011
C	-1.66565	-1.42844	-0.39107
H	-2.73042	2.17869	0.56721
H	-4.32426	0.30724	0.76879
H	-3.66362	-1.97876	0.15971
H	-1.37587	-2.43283	-0.66325
C	1.07464	1.06392	-0.52947
H	2.16856	1.00158	-1.34256
C	0.63357	-0.30901	-1.01175
H	0.60443	-0.33499	-2.10240
C	2.15931	0.82591	0.54154
H	2.64982	1.73721	0.86698
C	1.85601	-1.13045	-0.52549
H	2.08383	-2.00697	-1.12091
C	2.87500	0.00812	-0.52105
H	3.94137	-0.18604	-0.49569
C	1.65916	-1.35751	0.95382
H	1.33268	-2.28919	1.38728
C	1.78469	-0.18601	1.57550
H	1.58508	0.01332	2.61595
N	-0.10426	1.80895	-0.34066
H	-0.06776	2.62729	0.23729

TS39

0	2		
C	-0.96779	-0.50604	-0.24224
C	-1.27800	0.84004	0.05447
C	-2.58364	1.23325	0.27565
C	-3.58186	0.27084	0.20971

C	-3.28533	-1.06095	-0.05527
C	-1.97768	-1.45854	-0.27064
H	-2.82265	2.26018	0.50765
H	-4.60723	0.56437	0.37461
H	-4.08128	-1.78805	-0.09309
H	-1.74554	-2.49259	-0.47518
C	0.90899	0.84497	-0.51170
H	0.84626	1.02260	-1.59745
C	0.45880	-0.55627	-0.32332
C	2.41939	0.86446	-0.21734
H	2.85513	1.85259	-0.37336
C	1.45942	-1.45016	0.04183
H	1.27462	-2.51472	-0.02723
C	2.91152	-0.16785	-1.21051
H	3.86818	-0.62760	-1.01604
C	2.30999	-0.99054	1.15988
H	2.45162	-1.65889	1.99634
C	2.73470	0.26816	1.11470
H	3.23483	0.79075	1.91205
N	-0.12207	1.61420	0.14515
H	-0.21545	2.57710	-0.12419
H	2.62488	-0.07348	-2.25153

TS40

0	2		
C	-0.87788	-0.47698	-0.23223
C	-1.33134	0.84642	-0.14791
C	-2.65668	1.14244	0.09471
C	-3.53431	0.08254	0.27821
C	-3.10010	-1.23086	0.20347
C	-1.76598	-1.51512	-0.06792
H	-3.00229	2.16344	0.15168
H	-4.57310	0.28956	0.48521
H	-3.80160	-2.03673	0.35195
H	-1.43070	-2.53889	-0.14011
C	0.88737	1.00131	-0.28639
C	0.55378	-0.39162	-0.66155
H	0.52407	-0.39185	-1.76163
C	2.16570	1.20281	0.21716
H	2.55334	2.21142	0.29970
C	1.82181	-1.20402	-0.32805
H	1.73482	-2.24751	-0.63102
C	2.87893	-0.43060	-1.09878
H	3.90905	-0.60908	-0.82727
C	2.22202	-1.01803	1.10199
H	2.26620	-1.80749	1.83283
C	2.49021	0.26484	1.32377
H	2.81495	0.67698	2.26806
N	-0.27168	1.74569	-0.33873

H	-0.31115	2.64226	0.10762
H	2.69705	-0.21084	-2.14472

TS41

0	2		
C	1.04801	0.55071	0.09867
C	1.28079	-0.80096	-0.18590
C	2.56524	-1.31072	-0.25778
C	3.61971	-0.43648	-0.05721
C	3.40397	0.91240	0.20645
C	2.11634	1.41291	0.28283
H	2.74322	-2.35305	-0.47392
H	4.63063	-0.81107	-0.10847
H	4.24654	1.56883	0.35647
H	1.94493	2.45765	0.49442
C	-0.96000	-0.59670	0.03648
C	-0.38559	0.74086	0.08227
C	-2.40405	-0.82633	-0.34652
H	-2.54542	-1.42350	-1.24900
C	-1.17976	1.83391	0.02566
H	-0.76659	2.82898	0.08066
C	-2.70929	-1.57090	0.94413
C	-2.59012	1.66138	-0.20978
H	-3.20643	2.54641	-0.26677
C	-3.15140	0.46325	-0.43580
H	-4.20146	0.39839	-0.68211
N	0.08012	-1.45308	-0.40407
H	0.01957	-2.43626	-0.21608
H	-3.47956	-1.19580	1.60362
H	-2.54595	-2.64158	0.95127
H	-1.32346	-0.96752	1.23749

TS42

0	2		
C	1.04554	0.55894	0.03762
C	1.29717	-0.79112	-0.25425
C	2.58113	-1.31429	-0.25553
C	3.61925	-0.45766	0.05026
C	3.38898	0.88646	0.34757
C	2.10835	1.40029	0.34223
H	2.76597	-2.35278	-0.48423
H	4.62977	-0.83616	0.05980
H	4.22361	1.52826	0.58244
H	1.93402	2.44108	0.57020
C	-0.91477	-0.49645	-0.41738
C	-0.37791	0.74391	-0.06734
C	-2.30094	-0.70954	-0.54220
H	-2.67100	-1.60147	-1.02420
C	-1.22102	1.84422	0.08405

H	-0.82182	2.81065	0.35040
C	-2.72510	-1.67965	1.38807
C	-2.57657	1.68002	-0.14973
H	-3.23636	2.53072	-0.07638
C	-3.10135	0.44524	-0.50206
H	-4.15819	0.35118	-0.69814
N	0.09994	-1.40927	-0.51497
H	-0.00844	-2.36711	-0.77704
H	-2.07055	-2.53915	1.37007
H	-3.78256	-1.88352	1.31154
H	-2.44065	-0.87898	2.05245

TS43

0	2		
C	1.06753	0.57164	0.06654
C	1.23414	-0.81789	0.06336
C	2.48995	-1.38828	-0.03806
C	3.58358	-0.54418	-0.14605
C	3.43082	0.83740	-0.15996
C	2.17110	1.40162	-0.05806
H	2.61616	-2.46023	-0.04378
H	4.57229	-0.97002	-0.22358
H	4.29925	1.47126	-0.24630
H	2.04916	2.47434	-0.06003
C	-0.98733	-0.48552	0.51929
C	-0.34596	0.82909	0.17927
C	-2.33740	-0.62727	-0.10013
C	-1.10411	1.93092	0.01706
H	-0.65419	2.89548	-0.16431
C	-2.89382	-1.97187	-0.24768
C	-2.52712	1.79808	-0.01491
H	-3.13863	2.68546	-0.06354
C	-3.11764	0.57308	-0.11385
H	-4.18421	0.49848	-0.26893
N	0.00779	-1.46932	0.12281
H	0.00182	-2.33014	0.63985
H	-2.26215	-2.83057	-0.10924
H	-3.92480	-2.10194	-0.52914
H	-2.27825	-1.16048	-1.20663
H	-1.13337	-0.52739	1.61505

TS44

0	2		
C	1.08484	0.56657	0.00421
C	1.23887	-0.82833	-0.01590
C	2.49132	-1.42246	-0.03006
C	3.59459	-0.59239	-0.03808
C	3.45995	0.79642	-0.03327
C	2.21067	1.38157	-0.01296

H	2.60277	-2.49582	-0.04077
H	4.58172	-1.02827	-0.04933
H	4.34319	1.41553	-0.04178
H	2.10793	2.45612	-0.00318
C	-0.96370	-0.43806	0.13222
C	-0.32594	0.82612	0.03651
C	-2.35868	-0.57958	-0.04174
C	-1.09948	1.97582	0.00044
H	-0.63693	2.95044	-0.02680
C	-2.96994	-1.93449	-0.08518
C	-2.47302	1.84790	-0.03039
H	-3.09180	2.73114	-0.05919
C	-3.08399	0.58782	-0.07134
H	-4.16025	0.52933	-0.14344
N	-0.00060	-1.42164	-0.04428
H	-0.14918	-2.37258	0.22925
H	-4.04418	-1.87650	-0.22945
H	-2.78702	-2.47376	0.84628
H	-2.55212	-2.53020	-0.89666
H	-1.04278	-0.47546	1.83821

TS45

0	2		
C	-0.83649	-0.36777	-0.03776
C	-1.35385	0.92066	0.14473
C	-2.71189	1.16435	0.10028
C	-3.55321	0.08754	-0.14446
C	-3.05370	-1.19312	-0.33284
C	-1.68683	-1.42841	-0.27232
H	-3.11035	2.15734	0.24148
H	-4.61822	0.25485	-0.19345
H	-3.73201	-2.00892	-0.52675
H	-1.29480	-2.42477	-0.41595
C	0.89284	1.15901	0.15398
C	0.61410	-0.27400	0.15369
H	0.83766	-0.75333	1.33809
C	2.12833	1.65513	-0.04973
H	2.33051	2.71409	-0.03427
C	1.66363	-1.26126	-0.31664
H	1.36320	-1.86786	-1.17278
C	1.64253	-2.00863	1.00554
C	2.97722	-0.59197	-0.56156
H	3.79448	-1.22408	-0.87703
C	3.17689	0.72149	-0.38751
H	4.16471	1.12375	-0.55867
N	-0.31059	1.80320	0.35641
H	-0.41067	2.79404	0.26550
H	2.55414	-2.10085	1.57906
H	0.89797	-2.78483	1.12755

TS46

0	2		
C	-0.84237	-0.32378	-0.24427
C	-1.36751	0.92697	0.12617
C	-2.73060	1.13130	0.27354
C	-3.56698	0.05508	0.04995
C	-3.06355	-1.19560	-0.31172
C	-1.70598	-1.39151	-0.46107
H	-3.12790	2.09442	0.55553
H	-4.63265	0.18530	0.15912
H	-3.74625	-2.01446	-0.47605
H	-1.31838	-2.35927	-0.74323
C	0.86006	1.15443	0.04374
C	0.57987	-0.17149	-0.30205
C	2.15826	1.64891	0.08126
H	2.36554	2.67453	0.34394
C	1.63460	-1.07148	-0.55213
H	1.42407	-2.02217	-1.01761
C	1.70439	-2.17925	1.36613
C	2.93343	-0.53948	-0.61655
H	3.75456	-1.18091	-0.89569
C	3.18085	0.77804	-0.26799
H	4.19620	1.14416	-0.28335
N	-0.32474	1.80021	0.29618
H	-0.41359	2.76405	0.54228
H	2.48090	-2.90794	1.18933
H	0.69184	-2.55085	1.41885
H	1.95203	-1.36116	2.02351

TS47

0	2		
C	-0.83061	0.29624	0.31258
C	-1.28765	-0.96218	-0.07841
C	-2.62145	-1.19622	-0.35191
C	-3.50543	-0.13506	-0.22023
C	-3.07215	1.11495	0.18942
C	-1.72643	1.32980	0.46782
H	-2.96937	-2.17202	-0.65416
H	-4.55129	-0.29272	-0.43500
H	-3.77923	1.92225	0.29574
H	-1.39422	2.30109	0.80447
C	0.95874	-1.20348	0.11460
C	0.63950	0.19152	0.59112
C	2.22510	-1.62166	-0.02243
H	2.46745	-2.63888	-0.28793
C	1.63282	1.16874	0.00463
C	1.23990	2.33043	-0.81298
C	2.99565	0.69432	0.02573

H	3.79595	1.41223	-0.08201
C	3.26896	-0.63123	0.07841
H	4.29642	-0.96121	0.08540
N	-0.23333	-1.85763	-0.10849
H	-0.27829	-2.71770	-0.61818
H	0.20318	2.59778	-0.89587
H	1.56978	2.29477	0.50495
H	1.97803	2.81635	-1.42705
H	0.79308	0.18441	1.67892

TS48

0	2		
C	-0.80581	0.32815	0.05209
C	-1.33662	-0.96881	0.00593
C	-2.69936	-1.20380	-0.06879
C	-3.53690	-0.10568	-0.10161
C	-3.03008	1.18996	-0.04962
C	-1.66838	1.41335	0.03630
H	-3.09463	-2.20751	-0.10120
H	-4.60337	-0.25745	-0.16538
H	-3.70891	2.02785	-0.07141
H	-1.29033	2.42041	0.09563
C	0.89445	-1.21430	0.04773
C	0.64157	0.18028	0.17238
C	2.17477	-1.72555	-0.03025
H	2.35633	-2.78840	-0.06799
C	1.71119	1.08758	-0.03285
C	1.46397	2.55459	-0.04458
C	2.97977	0.56014	-0.11529
H	3.81950	1.23211	-0.21309
C	3.21421	-0.81802	-0.08991
H	4.22887	-1.17930	-0.15440
N	-0.30301	-1.87308	0.04463
H	-0.40674	-2.86152	-0.05816
H	0.80862	2.83997	-0.86644
H	0.98095	2.86940	0.88160
H	2.39351	3.10616	-0.14670
H	0.62069	0.24259	1.86599

Table S8. Cartesian coordinates for reactions, intermediate, and products involved in the NSO-HETs formation routes, x coordinate, y coordinate, and z coordinate.

methane

0	1		
C	0.00000	0.00000	0.00000
H	0.62658	0.62658	0.62658
H	-0.62658	0.62658	-0.62658
H	0.62658	-0.62658	-0.62658
H	-0.62658	-0.62658	0.62658

cyclopenthiophene

C	-1.16725	-0.28459	-0.00006
C	-0.73100	0.98127	-0.00008
C	0.72544	0.98539	0.00001
C	1.16884	-0.27801	0.00008
H	-2.19341	-0.61119	-0.00010
H	-1.34689	1.86577	-0.00015
H	1.33632	1.87336	0.00001
H	2.19684	-0.59880	0.00015
C	0.00340	-1.20478	0.00005
H	0.00521	-1.86239	0.87271
H	0.00531	-1.86245	-0.87257

benzofuran

0	1		
C	-0.25662	0.74965	0.00003
C	-0.24993	-0.64274	-0.00009
C	0.90052	-1.40459	0.00000
C	2.09411	-0.71056	0.00021
C	2.12256	0.68657	0.00032
C	0.95950	1.42778	0.00024
C	-1.64260	1.11881	-0.00011
C	-2.32757	-0.03700	-0.00028
H	0.86190	-2.48158	-0.00010
H	3.02384	-1.25784	0.00028
H	3.07548	1.19257	0.00048
H	0.99211	2.50624	0.00033
H	-2.06426	2.10622	-0.00007
H	-3.37850	-0.25386	-0.00043
O	-1.51381	-1.11741	-0.00030

benzothiophene

0	1		
C	0.10749	0.84295	0.00002
C	-0.06643	-0.54844	-0.00003
C	1.01983	-1.41581	0.00010
C	2.28740	-0.87721	0.00028
C	2.47832	0.50674	0.00033

C	1.40326	1.36367	0.00021
C	-1.14456	1.53488	-0.00013
C	-2.19688	0.69446	-0.00028
H	0.87454	-2.48485	0.00007
H	3.14322	-1.53429	0.00038
H	3.48149	0.90385	0.00048
H	1.55304	2.43276	0.00025
S	-1.74406	-0.96804	-0.00024
H	-1.23756	2.60816	0.00002
H	-3.24024	0.95567	-0.00042

indole

0	1		
C	-0.24772	0.74366	-0.00003
C	-0.24723	-0.66258	-0.00011
C	0.92614	-1.40696	-0.00023
C	2.11545	-0.71413	0.00005
C	2.14022	0.68643	0.00015
C	0.97480	1.41772	-0.00008
C	-1.61367	1.15665	-0.00005
C	-2.36655	0.02680	-0.00004
H	0.90871	-2.48635	-0.00034
H	3.04659	-1.25943	0.00008
H	3.09136	1.19593	0.00035
H	1.00343	2.49684	-0.00002
H	-1.98803	2.16332	-0.00016
H	-3.43470	-0.08750	0.00007
N	-1.55070	-1.06988	0.00023
H	-1.86108	-2.01923	0.00035

cyclopentadienyl

0	2		
C	-1.16093	-0.35205	-0.00005
C	-0.73439	0.93880	-0.00007
C	0.73223	0.94038	0.00000
C	1.16170	-0.34965	0.00008
H	0.00251	-2.25529	0.00011
H	-2.17824	-0.69971	-0.00011
H	-1.35122	1.82145	-0.00014
H	1.34718	1.82435	0.00000
H	2.17978	-0.69506	0.00016
C	0.00139	-1.17677	0.00004

dibenzofuran

0	1		
C	-0.72017	0.52910	0.00017
C	-1.08575	-0.81454	0.00012
C	-2.39655	-1.23882	0.00005
C	-3.36751	-0.25292	-0.00006

C	-3.03052	1.09981	-0.00012
C	-1.70932	1.50266	-0.00002
H	-2.64488	-2.28749	0.00006
H	-4.40776	-0.53913	-0.00013
H	-3.81499	1.84013	-0.00029
H	-1.45324	2.55091	-0.00005
O	-0.00006	-1.62888	0.00006
C	1.08571	-0.81460	-0.00005
C	3.03055	1.09980	0.00001
H	3.81505	1.84009	0.00013
C	3.36748	-0.25293	-0.00010
C	4.40771	-0.53923	-0.00010
C	1.70940	1.50265	0.00009
H	2.39650	-1.23887	-0.00009
H	2.64499	-2.28751	-0.00014
C	1.45325	2.55090	0.00014

dibenzothiophene

0	1		
C	0.72169	0.56941	0.00000
C	1.24370	-0.72759	0.00002
C	2.61234	-0.95511	0.00001
C	3.46082	0.13291	-0.00001
C	2.95564	1.43128	-0.00001
C	1.59552	1.65193	0.00000
C	-0.72167	0.56938	0.00003
C	-1.24367	-0.72766	0.00007
H	3.00562	-1.95964	0.00000
H	4.52798	-0.02630	-0.00004
H	3.63548	2.26884	-0.00002
H	1.20899	2.65984	0.00002
S	0.00008	-1.94481	0.00000
C	-2.61227	-0.95513	0.00000
C	-1.59564	1.65187	-0.00004
C	-3.46088	0.13277	0.00006
H	-1.20923	2.65984	-0.00003
C	-2.95574	1.43123	-0.00002
H	-3.63561	2.26880	-0.00004
H	-4.52802	-0.02642	0.00003

carbazole

0	1		
C	-0.71966	0.50034	0.00011
C	-1.11970	-0.84533	-0.00002
C	-2.45705	-1.21169	-0.00013
C	-3.39667	-0.20072	-0.00013
C	-3.01901	1.14266	0.00001
C	-1.68573	1.49884	0.00014
H	-2.75597	-2.24877	-0.00015

H	-4.44522	-0.45600	-0.00025
H	-3.77920	1.90787	0.00004
H	-1.39665	2.53905	0.00029
C	1.11968	-0.84526	0.00012
C	0.71962	0.50039	0.00018
C	2.45698	-1.21169	0.00008
C	1.68579	1.49879	-0.00004
H	1.39693	2.53905	-0.00020
C	3.01905	1.14259	-0.00026
H	3.77916	1.90787	-0.00043
C	3.39668	-0.20076	0.00001
H	4.44521	-0.45609	-0.00003
N	-0.00001	-1.63680	0.00010
H	0.00004	-2.63553	-0.00060
H	2.75576	-2.24881	0.00019

4-methyl-dibenzofuran

0	1		
C	-1.06760	0.59868	0.00006
C	-1.19582	-0.78785	0.00005
C	-2.41367	-1.43262	0.00001
C	-3.54064	-0.62971	-0.00004
C	-3.44225	0.76072	-0.00005
C	-2.21054	1.38619	-0.00001
H	-2.47694	-2.50846	0.00002
H	-4.51576	-1.09138	-0.00007
H	-4.34294	1.35438	-0.00011
H	-2.13957	2.46293	0.00000
O	0.01443	-1.40144	0.00003
C	0.35103	0.84921	0.00004
C	2.30906	-0.62660	-0.00001
C	2.52408	1.80062	-0.00002
H	3.17708	2.65942	-0.00004
C	3.08378	0.52290	-0.00003
H	4.15903	0.41966	-0.00005
C	1.15613	1.98054	0.00002
H	0.72664	2.97018	0.00006
C	0.94337	-0.40850	0.00001
C	2.87632	-2.00558	-0.00003
H	3.96149	-1.97646	0.00002
H	2.54798	-2.56339	0.87450
H	2.54806	-2.56333	-0.87463

4-methyl-dibenzothiophene

0	1		
C	-1.08699	0.58790	0.00070
C	-1.37227	-0.78092	0.00053
C	-2.67921	-1.24596	0.00021
C	-3.70645	-0.32471	-0.00036

C	-3.43792	1.04242	-0.00066
C	-2.13803	1.49928	-0.00013
C	0.33372	0.84373	0.00045
C	1.07498	-0.33959	0.00039
H	-2.88922	-2.30413	0.00037
H	-4.72881	-0.66966	-0.00046
H	-4.25463	1.74721	-0.00141
H	-1.93450	2.55944	-0.00033
S	0.06679	-1.75965	0.00003
C	2.46785	-0.35068	-0.00014
C	1.00310	2.06307	0.00036
C	3.09941	0.87960	-0.00031
H	0.44990	2.98982	0.00025
C	2.38024	2.07257	-0.00008
C	3.22109	-1.63860	-0.00043
H	2.97409	-2.23669	0.87611
H	2.97435	-2.23603	-0.87747
H	2.91114	3.01179	0.00011
H	4.17914	0.91070	-0.00076
H	4.29289	-1.46670	-0.00002

4-methyl-carbazole

0	1		
C	0.79106	0.32913	0.00016
C	1.33100	-0.96980	0.00006
C	2.69782	-1.20098	-0.00008
C	3.53308	-0.10225	-0.00012
C	3.02086	1.19459	-0.00004
C	1.65858	1.41715	0.00010
H	3.09641	-2.20403	-0.00011
H	4.60185	-0.25157	-0.00026
H	3.69949	2.03308	-0.00010
H	1.27885	2.42538	0.00022
C	-0.89698	-1.20262	0.00010
C	-0.64302	0.17818	0.00012
C	-2.18382	-1.71954	0.00006
H	-2.36547	-2.78315	0.00004
C	-1.70818	1.08244	-0.00002
C	-1.47369	2.55655	0.00000
C	-2.98906	0.56034	-0.00016
H	-3.83011	1.23775	-0.00031
C	-3.22316	-0.81449	-0.00005
H	-4.24029	-1.17538	-0.00013
N	0.29955	-1.87113	-0.00007
H	0.40230	-2.86459	0.00031
H	-0.90661	2.86450	0.87736
H	-0.90614	2.86443	-0.87706
H	-2.41410	3.09932	-0.00026

2-methyl-dibenzothiophene

0	1		
C	0.76556	0.43367	0.00000
C	1.42396	-0.80467	0.00000
C	2.80621	-0.90749	0.00001
C	3.55622	0.24928	0.00001
C	2.92861	1.49022	0.00000
C	1.55279	1.58746	0.00000
C	-0.67757	0.27236	0.00001
C	-1.03743	-1.08211	-0.00001
H	3.28418	-1.87466	0.00001
H	4.63345	0.18945	0.00003
H	3.52280	2.39075	0.00000
H	1.09991	2.56269	-0.00002
S	0.33024	-2.15065	-0.00001
C	-2.35827	-1.50264	-0.00001
C	-1.69484	1.24028	0.00002
C	-3.34362	-0.54140	0.00001
C	-3.00835	0.80772	0.00003
H	-3.79587	1.54655	0.00002
H	-4.38167	-0.83586	0.00002
H	-2.60328	-2.55305	-0.00005
C	-1.41409	2.70777	-0.00003
H	-0.84687	3.00758	-0.87930
H	-2.34495	3.26665	0.00001
H	-0.84678	3.00760	0.87918

2-methyl-dibenzofuran

0	1		
C	-0.79236	0.36612	-0.00097
C	-1.30424	-0.93114	0.00001
C	-2.65090	-1.21888	0.00131
C	-3.51570	-0.13952	0.00130
C	-3.03783	1.16902	-0.00029
C	-1.68133	1.43512	-0.00163
H	-3.00255	-2.23761	0.00225
H	-4.58010	-0.31569	0.00243
H	-3.74018	1.98777	-0.00055
H	-1.32847	2.45378	-0.00356
O	-0.31683	-1.85865	-0.00044
C	0.85213	-1.17181	-0.00097
C	2.99495	0.49398	0.00105
H	3.86295	1.13657	0.00218
C	3.17573	-0.88765	0.00020
H	4.17812	-1.28686	0.00046
C	1.73604	1.07160	0.00052
C	2.10037	-1.75470	-0.00056
H	2.22000	-2.82540	-0.00049
C	0.64273	0.20428	-0.00103

C	1.55135	2.55252	0.00083
H	1.01186	2.88193	-0.88612
H	0.97874	2.87674	0.86854
H	2.50862	3.06441	0.01973

2-methyl-carbazole

0	1		
C	2.49487	1.83740	0.00004
C	3.09416	0.57594	0.00003
C	2.35435	-0.59142	0.00000
C	0.97170	-0.43665	-0.00001
C	0.34448	0.81648	0.00005
C	1.12274	1.96828	0.00006
H	3.11904	2.71732	0.00003
H	4.17237	0.50569	0.00008
H	0.66206	2.94440	0.00004
C	-1.23282	-0.83026	0.00005
C	-2.48633	-1.42351	0.00009
C	-3.58739	-0.59131	0.00004
C	-3.44850	0.79726	-0.00006
C	-2.19740	1.37989	-0.00011
C	-1.07244	0.56463	-0.00004
H	-2.60024	-2.49681	0.00014
H	-4.57569	-1.02472	0.00009
H	-4.33015	1.41881	-0.00009
H	-2.09350	2.45450	-0.00016
N	0.00817	-1.41418	-0.00004
H	0.18339	-2.39752	0.00013
C	2.97424	-1.94917	-0.00009
H	2.67822	-2.52277	0.87861
H	2.67950	-2.52204	-0.87968
H	4.05784	-1.88298	0.00077

IM1

0	2		
C	1.09716	0.33064	-0.73460
C	1.32196	-0.77666	0.06282
C	2.47109	-0.93013	0.80923
C	3.41573	0.08449	0.73614
C	3.20822	1.20563	-0.05238
C	2.04089	1.33297	-0.79527
H	2.62073	-1.80485	1.42122
H	4.32675	-0.00406	1.30809
H	3.95644	1.98171	-0.08807
H	1.87816	2.20511	-1.41078
O	0.31003	-1.67049	0.02343
C	-1.58705	0.27084	1.23507
C	-3.12282	-0.25325	-0.44530
C	-2.47792	1.26496	1.12950

H	-0.74982	0.21716	1.91040
C	-3.43584	0.93773	0.08065
H	-3.64255	-0.76250	-1.24015
H	-2.49704	2.16772	1.71794
H	-4.26322	1.56381	-0.21195
C	-1.91532	-0.79405	0.24311
H	-2.15529	-1.72679	0.76152
C	-0.77110	-1.09844	-0.71970
H	-1.09400	-1.85466	-1.42980
C	-0.22200	0.15504	-1.41255
H	-0.10528	0.00347	-2.48424

IM2

0	2		
C	-0.86903	-0.16710	-0.25844
C	-1.81404	0.80407	0.02028
C	-3.13770	0.52353	0.27304
C	-3.51534	-0.80947	0.22539
C	-2.59542	-1.80448	-0.07192
C	-1.26613	-1.48790	-0.31848
H	-3.84053	1.31131	0.48975
H	-4.54414	-1.07337	0.41569
H	-2.91683	-2.83316	-0.11539
H	-0.56299	-2.26697	-0.56702
O	-1.28825	2.05578	-0.00737
C	0.45270	0.51324	-0.47215
H	0.80995	0.36470	-1.49690
C	1.59546	0.05602	0.46329
H	1.23605	0.15626	1.49139
C	2.08235	-1.33572	0.22698
C	2.83134	0.87216	0.27061
H	1.46853	-2.21482	0.31521
C	3.38989	-1.31028	-0.05613
H	2.85605	1.94477	0.36665
C	3.85737	0.06982	-0.03388
H	4.01296	-2.16646	-0.25792
H	4.87191	0.37738	-0.22789
C	0.05264	1.93173	-0.22798
H	0.53054	2.82043	-0.59823

IM3

0	2		
C	0.77939	0.50506	-0.51194
C	1.00630	-0.82759	-0.20928
C	2.21894	-1.27761	0.26976
C	3.22532	-0.33808	0.43905
C	3.02142	1.00043	0.13837
C	1.78940	1.42785	-0.34027
H	2.37013	-2.32030	0.49741

H	4.18720	-0.65953	0.80862
H	3.82226	1.70989	0.27543
H	1.62680	2.46990	-0.57380
O	-0.05845	-1.62392	-0.43947
C	-1.12419	-0.82256	-0.94063
H	-1.39289	-1.20452	-1.92140
C	-0.63573	0.64409	-0.96234
H	-0.72585	1.08964	-1.95035
C	-2.33936	-0.79391	0.00419
H	-3.00178	-1.64690	-0.08074
C	-1.62680	1.33920	0.01277
H	-1.64953	2.42015	-0.06226
C	-2.87912	0.57277	-0.30844
H	-3.86673	0.89619	-0.01266
C	-1.37069	0.76629	1.38785
H	-0.85577	1.27450	2.18706
C	-1.79146	-0.49848	1.38322
H	-1.68673	-1.22229	2.17455

IM4

0	2		
C	0.81099	0.48682	-0.45403
C	1.12828	-0.84646	-0.14361
C	2.38676	-1.23301	0.25405
C	3.35893	-0.24421	0.34263
C	3.06924	1.08644	0.05627
C	1.79722	1.46321	-0.33406
H	2.59818	-2.26167	0.49709
H	4.35871	-0.51597	0.64448
H	3.84713	1.82920	0.13935
H	1.57169	2.49487	-0.55671
O	0.06897	-1.67627	-0.23628
C	-1.01635	-0.92572	-0.76765
H	-1.23533	-1.33452	-1.75696
C	-0.57025	0.50431	-0.78224
C	-2.31286	-0.85166	0.05970
H	-2.92140	-1.74850	0.03143
C	-1.71210	1.30686	-0.20104
H	-1.76425	2.35915	-0.45446
C	-1.60554	0.96183	1.27423
H	-1.17585	1.60895	2.02160
C	-1.95528	-0.31445	1.41995
H	-1.88866	-0.90565	2.31760
C	-2.89419	0.41220	-0.59086
H	-3.82512	0.72162	-0.12458
H	-3.02587	0.32560	-1.66857

IM5

0 2

C	0.81756	0.54092	-0.42403
C	1.08173	-0.80715	-0.24037
C	2.31358	-1.28349	0.14908
C	3.31130	-0.34417	0.36331
C	3.07556	1.01135	0.18325
C	1.82476	1.46103	-0.21899
H	2.48523	-2.33937	0.28048
H	4.29007	-0.67718	0.67287
H	3.87131	1.71928	0.35426
H	1.64509	2.51576	-0.36529
O	-0.00060	-1.59345	-0.48831
C	-1.03744	-0.77973	-0.87341
C	-0.59917	0.66712	-0.87241
H	-0.67377	1.13930	-1.85386
C	-2.33416	-0.86105	-0.09872
H	-2.95425	-1.73009	-0.28465
C	-1.67854	1.28419	0.06800
H	-1.72560	2.36774	0.04104
C	-1.50716	0.64977	1.42343
H	-1.04072	1.12286	2.27168
C	-1.89894	-0.61894	1.33126
H	-1.79928	-1.38689	2.08055
C	-2.90348	0.51683	-0.44153
H	-3.80344	0.74321	0.12316
H	-3.08334	0.65291	-1.50689

IM6

0	2		
C	-0.95188	0.61767	0.07470
C	-1.22086	-0.73559	0.26908
C	-2.49483	-1.25356	0.17225
C	-3.51591	-0.36897	-0.13773
C	-3.27011	0.98486	-0.34007
C	-1.98441	1.48647	-0.23836
H	-2.67823	-2.30393	0.32910
H	-4.52519	-0.74184	-0.22202
H	-4.08862	1.64616	-0.57632
H	-1.78886	2.53637	-0.39436
O	-0.11990	-1.46707	0.53871
C	0.96336	-0.56186	0.71943
H	1.16657	-0.48940	1.79355
C	0.47493	0.76751	0.22689
C	1.35847	1.74584	0.00987
H	1.05734	2.73590	-0.29633
C	2.77084	1.42323	0.13354
H	3.48701	2.23131	0.15782
C	3.19929	0.15636	0.14230
H	4.25664	-0.06194	0.15270
C	2.02224	-1.39725	-1.39343

H	2.73767	-1.12835	-2.15284
H	1.18935	-2.03030	-1.65388
C	2.23468	-0.99848	0.02366
H	2.63073	-1.85480	0.58019

IM7

0	2		
C	0.82173	0.36144	-0.18936
C	1.30121	-0.92645	-0.01702
C	2.63228	-1.21764	0.16236
C	3.51109	-0.14317	0.17281
C	3.06013	1.15700	0.00385
C	1.70842	1.41715	-0.18673
H	2.96825	-2.23327	0.29250
H	4.56461	-0.32698	0.31655
H	3.76565	1.97273	0.01662
H	1.35828	2.42756	-0.33244
O	0.30822	-1.86342	-0.03864
C	-0.87214	-1.19682	-0.13614
C	-3.00993	0.52845	0.15141
H	-3.85330	1.20187	0.18177
C	-3.19344	-0.78495	-0.01099
H	-4.19311	-1.17704	-0.12561
C	-1.53768	2.54308	0.05541
H	-1.94789	2.90989	-0.87344
H	-0.97415	3.22493	0.67077
C	-1.63209	1.10470	0.39093
C	-2.08059	-1.72119	0.02740
H	-2.23327	-2.77182	0.20989
H	-1.38528	0.96766	1.45058
C	-0.65147	0.25767	-0.42615
H	-0.88063	0.43615	-1.48470

IM8

0	2		
C	1.07771	0.59923	0.02660
C	1.25259	-0.77233	-0.13444
C	2.48452	-1.38817	-0.14353
C	3.58573	-0.56689	0.01889
C	3.44377	0.81075	0.18259
C	2.19687	1.40603	0.18857
H	2.57797	-2.45425	-0.27128
H	4.57318	-1.00131	0.01869
H	4.32513	1.42051	0.30643
H	2.09685	2.47297	0.31527
O	0.05691	-1.39867	-0.27861
C	-0.34739	0.80727	-0.02682
C	-2.31511	-0.77062	-0.33002
H	-2.48547	-1.22288	-1.31660

C	-2.56989	1.72889	-0.04712
H	-3.23219	2.57937	0.01879
C	-3.11278	0.49255	-0.23278
H	-4.18524	0.38726	-0.30812
C	-1.18563	1.94151	0.06217
H	-0.78180	2.92935	0.20840
C	-0.88539	-0.43257	-0.21151
C	-2.75137	-1.80972	0.70822
H	-3.80233	-2.05415	0.57456
H	-2.16932	-2.72135	0.60388
H	-2.61383	-1.42173	1.71395

IM9

0	2		
C	1.07159	0.59562	-0.13100
C	1.18004	-0.79783	-0.06483
C	2.38959	-1.43418	0.10542
C	3.51892	-0.63504	0.21106
C	3.43534	0.75311	0.15441
C	2.21348	1.37825	-0.00959
H	2.44612	-2.50928	0.15618
H	4.48286	-1.10292	0.33918
H	4.33380	1.34386	0.23892
H	2.14598	2.45463	-0.04998
O	-0.01129	-1.42532	-0.15191
C	-0.32401	0.87875	-0.24533
C	-2.32342	-0.59677	0.04456
C	-2.46390	1.83563	0.13603
H	-3.08692	2.69870	0.30838
C	-3.03285	0.53896	0.25586
H	-4.06547	0.45926	0.56494
C	-1.09146	1.99524	-0.03991
H	-0.63585	2.96683	0.07883
C	-0.96694	-0.43295	-0.53683
H	-1.06481	-0.54150	-1.63330
C	-2.85731	-1.97142	0.20254
H	-3.87362	-1.95751	0.58473
H	-2.85242	-2.50460	-0.74963
H	-2.23390	-2.54926	0.88270

IM10

0	2		
C	0.82895	0.36912	0.13192
C	1.35222	-0.91126	-0.08321
C	2.69929	-1.17794	-0.17020
C	3.55549	-0.09937	-0.02769
C	3.06774	1.18912	0.19566
C	1.71286	1.43813	0.27777
H	3.06086	-2.17926	-0.33829

H	4.62060	-0.25944	-0.08794
H	3.76633	2.00388	0.30597
H	1.34765	2.43810	0.45375
O	0.36258	-1.82599	-0.18598
C	-0.80126	-1.14270	-0.03526
C	-3.01754	0.44579	0.33740
H	-3.89675	1.05589	0.48678
C	-3.15851	-0.89074	0.14673
H	-4.14927	-1.31969	0.15334
C	-1.70457	1.16154	0.32084
C	-2.05419	-1.74840	-0.05649
H	-2.16634	-2.80832	-0.20602
H	-1.58520	1.68217	1.27975
C	-0.58480	0.19977	0.15556
C	-1.69699	2.24342	-0.76681
H	-0.76108	2.79616	-0.76054
H	-1.81917	1.79144	-1.74745
H	-2.51024	2.94816	-0.60879

IM11

0	2		
C	-0.80876	0.35030	0.20531
C	-1.28833	-0.93764	0.02396
C	-2.61170	-1.22553	-0.21128
C	-3.48657	-0.15023	-0.26544
C	-3.03856	1.14823	-0.07784
C	-1.69608	1.40596	0.17164
H	-2.94319	-2.24157	-0.34980
H	-4.53317	-0.33119	-0.45547
H	-3.74003	1.96652	-0.11941
H	-1.36406	2.41823	0.34070
O	-0.30592	-1.88034	0.10335
C	0.87508	-1.21339	0.20545
C	2.94325	0.52838	-0.24629
H	3.76704	1.16757	-0.53198
C	3.17016	-0.86328	-0.17807
H	4.16001	-1.25004	-0.35680
C	1.72811	1.09719	-0.01718
C	2.08640	-1.75202	-0.04604
H	2.19615	-2.80923	-0.22681
C	0.65094	0.22007	0.54392
H	0.71887	0.32585	1.64576
C	1.47342	2.55422	-0.14659
H	0.70661	2.75534	-0.89488
H	1.11997	2.98411	0.79336
H	2.37505	3.08366	-0.44076

IM12

0	2

C	1.12842	0.56996	-0.55120
C	1.56299	-0.64101	0.04429
C	2.81782	-0.75853	0.59948
C	3.67145	0.33757	0.56897
C	3.26638	1.53940	-0.01099
C	2.01352	1.66475	-0.56489
C	-0.16770	0.50395	-1.04913
C	-0.86315	-0.79315	-0.85028
H	3.13569	-1.68523	1.05146
H	4.65706	0.25482	0.99964
H	3.94409	2.37890	-0.02366
H	1.69909	2.59559	-1.01148
S	0.34485	-1.88593	-0.02716
C	-2.16286	-0.66661	-0.03933
H	-2.54891	-1.67589	0.13837
C	-2.00439	0.04229	1.26488
C	-3.20902	0.14186	-0.73631
H	-1.29939	-0.26222	2.01984
C	-2.85083	1.07614	1.31285
H	-3.57953	-0.07920	-1.72386
C	-3.60265	1.13816	0.06461
H	-2.96610	1.76640	2.13238
H	-4.35714	1.87425	-0.16142
H	-1.11307	-1.25571	-1.80729
H	-0.68928	1.33456	-1.49706

IM13

0	2		
C	-0.89709	-0.41770	-0.50437
C	-1.54523	0.68470	0.03950
C	-2.81693	0.58328	0.57819
C	-3.44431	-0.64991	0.56951
C	-2.81180	-1.75926	0.02844
C	-1.53920	-1.64260	-0.50655
C	0.48171	-0.13674	-1.02452
C	0.66195	1.33762	-0.91490
H	-3.31030	1.44809	0.99369
H	-4.43577	-0.74458	0.98478
H	-3.31237	-2.71481	0.02289
H	-1.04871	-2.50855	-0.92801
S	-0.58469	2.14600	-0.05465
C	1.56624	-0.92615	-0.25183
H	1.31137	-1.98879	-0.32939
C	2.94825	-0.71106	-0.76973
C	1.68286	-0.54261	1.18577
H	3.23896	-0.88897	-1.79219
C	3.73385	-0.28421	0.22553
H	0.85440	-0.55631	1.87456
C	2.94309	-0.17750	1.44653

H	4.78497	-0.05608	0.15315
H	3.32706	0.15060	2.39861
H	0.55331	-0.45136	-2.07087
H	1.61177	1.82747	-1.04223

IM14

0	2		
C	0.80565	0.48906	-0.51419
C	1.16380	-0.77426	-0.06155
C	2.43485	-1.02210	0.43026
C	3.35657	0.01087	0.45692
C	3.01474	1.27349	-0.00054
C	1.73783	1.51037	-0.48443
C	-0.60817	0.61110	-0.98190
C	-1.30714	-0.76424	-0.84083
H	2.70527	-2.00538	0.78267
H	4.35044	-0.17430	0.83475
H	3.74127	2.07074	0.01884
H	1.46843	2.49420	-0.84147
S	-0.10452	-1.97246	-0.21252
C	-2.85567	0.90792	-0.36635
H	-3.78973	1.39151	-0.11850
C	-2.49754	-0.47286	0.10140
C	-1.51361	1.53116	-0.11507
H	-3.26708	-1.23577	0.09976
C	-1.91950	-0.10256	1.44917
H	-1.39049	2.59004	-0.31210
C	-1.33611	1.08961	1.32097
H	-1.91745	-0.73753	2.31960
H	-0.76130	1.61480	2.06644
H	-1.67335	-1.12355	-1.79689
H	-0.64789	0.94926	-2.01611

IM15

0	2		
C	-0.83929	-0.53039	-0.38364
C	-1.31309	0.76177	-0.06829
C	-2.63613	0.97584	0.25937
C	-3.50549	-0.10612	0.28549
C	-3.05208	-1.38975	0.00016
C	-1.72946	-1.60769	-0.32615
C	0.54218	-0.54367	-0.67632
C	1.16698	0.81387	-0.74293
H	-2.98964	1.96562	0.50344
H	-4.54291	0.05308	0.53566
H	-3.74117	-2.21943	0.03130
H	-1.37725	-2.60235	-0.55455
S	-0.05013	1.97764	-0.06444
C	2.87157	-0.76522	-0.69065

H	3.78557	-1.19513	-0.29047
C	2.51548	0.57007	-0.02397
C	1.61742	-1.48378	-0.19196
H	3.23215	1.37760	-0.12352
C	2.20738	0.10154	1.37366
H	1.50121	-2.53500	-0.42925
C	1.67557	-1.11614	1.28094
H	2.29299	0.70610	2.26062
H	1.22150	-1.69187	2.07093
H	1.36504	1.12214	-1.77115
H	2.92818	-0.70487	-1.77628

IM16

0	2		
C	-0.83301	-0.51716	-0.43167
C	-1.26073	0.76029	-0.08304
C	-2.56159	0.99998	0.32370
C	-3.44309	-0.06548	0.39161
C	-3.03205	-1.34441	0.04898
C	-1.73000	-1.56807	-0.36941
C	0.58654	-0.57549	-0.89437
C	1.18737	0.79247	-0.68973
H	-2.88327	1.99554	0.58738
H	-4.45895	0.10489	0.71356
H	-3.72838	-2.16661	0.10488
H	-1.41308	-2.56325	-0.64615
S	0.00540	1.97517	-0.23125
C	2.90180	-0.77161	-0.54176
H	3.77546	-1.18891	-0.04871
C	2.50248	0.58781	0.03398
C	1.60342	-1.46131	-0.11879
H	3.21975	1.39771	-0.03109
C	2.06715	0.16771	1.42400
H	1.51586	-2.52273	-0.32661
C	1.53256	-1.04811	1.32753
H	2.06754	0.81392	2.28649
H	1.02752	-1.59763	2.10444
H	0.61689	-0.86359	-1.95030
H	3.04929	-0.75191	-1.62019

IM17

0	2		
C	-1.09357	0.59394	0.05377
C	-1.39156	-0.76992	0.02061
C	-2.70311	-1.20943	-0.04502
C	-3.72016	-0.27248	-0.08934
C	-3.43608	1.08740	-0.06082
C	-2.12702	1.52031	0.01158
C	0.32951	0.85324	0.11441

C	1.11656	-0.39535	0.40201
H	-2.92872	-2.26422	-0.06355
H	-4.74532	-0.60548	-0.14165
H	-4.24047	1.80548	-0.08834
H	-1.90536	2.57639	0.04407
S	0.02383	-1.80050	0.03581
C	2.43092	-0.42772	-0.37160
C	0.99214	2.00936	-0.01534
C	3.13271	0.87726	-0.11211
H	0.46746	2.94060	-0.16885
C	2.44021	2.00974	0.03978
H	1.34555	-0.41910	1.47178
C	3.24766	-1.61579	-0.03354
H	3.90729	-1.60030	0.82044
H	3.07044	-2.55762	-0.52748
H	2.95667	2.95034	0.15979
H	4.21263	0.88730	-0.12813
H	2.16935	-0.46884	-1.43733

IM18

0	2		
C	-0.82762	0.43878	-0.33721
C	-1.40599	-0.77622	0.00984
C	-2.76390	-0.88888	0.24359
C	-3.55155	0.24581	0.13734
C	-2.98762	1.46704	-0.19394
C	-1.62561	1.56257	-0.43384
C	0.63855	0.34918	-0.62210
C	1.09079	-1.00240	-0.14401
H	-3.20227	-1.83743	0.51188
H	-4.61217	0.17487	0.32297
H	-3.60887	2.34609	-0.26471
H	-1.18594	2.51437	-0.69383
S	-0.24740	-2.09256	0.12192
C	2.38953	-1.27452	0.00001
C	1.52110	1.45875	-0.06212
C	3.34836	-0.20811	-0.22115
C	2.96790	1.07335	-0.27295
H	3.69829	1.86366	-0.35797
H	4.39468	-0.46485	-0.29546
H	2.73771	-2.25283	0.29263
H	1.30887	2.37367	-0.61371
C	1.34234	1.70145	1.39323
H	1.27604	0.85975	2.06568
H	1.54892	2.67292	1.81234
H	0.78538	0.34993	-1.71063

IM19

0	2

C	1.08914	0.58644	-0.01389
C	1.43227	-0.76561	0.08022
C	2.75422	-1.18498	0.04092
C	3.74120	-0.23097	-0.09426
C	3.41636	1.12150	-0.18927
C	2.10218	1.53186	-0.15019
C	-0.33819	0.78809	0.05096
C	-1.01770	-0.39646	0.18597
H	3.00524	-2.23164	0.11441
H	4.77529	-0.53716	-0.12654
H	4.20329	1.85203	-0.29444
H	1.85833	2.58071	-0.22416
S	0.02645	-1.77108	0.24044
C	-2.49948	-0.51689	0.28527
C	-1.04038	2.01333	-0.00209
C	-3.13654	0.83286	0.22626
H	-0.51121	2.94612	-0.10881
C	-2.44068	1.99200	0.08810
C	-3.08260	-1.44723	-0.78361
H	-2.85216	-1.07229	-1.77721
H	-2.67671	-2.45209	-0.69224
H	-2.98042	2.92644	0.04698
H	-4.21479	0.85749	0.29203
H	-2.74531	-0.96780	1.25631
H	-4.16345	-1.50830	-0.67950

IM20

0	2		
C	-1.11097	0.58653	0.12317
C	-1.36695	-0.78310	-0.03716
C	-2.66041	-1.25601	-0.16401
C	-3.70998	-0.35342	-0.12871
C	-3.47170	1.00641	0.03211
C	-2.17983	1.47752	0.15466
C	0.29527	0.87623	0.17890
C	1.08791	-0.34731	0.48707
H	-2.84972	-2.31075	-0.28851
H	-4.72296	-0.71338	-0.22160
H	-4.30046	1.69613	0.06714
H	-1.99548	2.53315	0.28650
S	0.08168	-1.76318	-0.11977
C	2.50784	-0.30682	0.04779
C	0.93046	2.06535	-0.06800
C	3.06442	0.90103	-0.21281
H	0.35035	2.96315	-0.22327
C	2.31345	2.10190	-0.21568
H	1.08523	-0.49053	1.57936
C	3.26954	-1.58142	0.04502
H	3.21370	-2.07490	1.01681

H	2.85564	-2.27904	-0.68450
H	2.80882	3.03623	-0.42496
H	4.11820	0.94832	-0.44815
H	4.31557	-1.41888	-0.19665

IM21

0	2		
C	0.79789	-0.43031	-0.12552
C	1.48045	0.78457	0.06205
C	2.86448	0.84221	0.09947
C	3.57852	-0.32789	-0.06298
C	2.92113	-1.54153	-0.26552
C	1.54636	-1.59957	-0.29725
C	-0.61592	-0.25517	-0.13564
C	-0.98299	1.06430	0.01368
H	3.37442	1.78145	0.24685
H	4.65678	-0.30112	-0.03831
H	3.49795	-2.44334	-0.40034
H	1.04391	-2.54077	-0.46338
S	0.38609	2.11966	0.21008
C	-2.31692	1.49209	-0.04097
C	-1.63359	-1.34012	-0.24773
C	-3.30707	0.52506	-0.30680
C	-3.00638	-0.79007	-0.44598
H	-3.79331	-1.50182	-0.64924
H	-4.33179	0.84936	-0.41044
H	-2.57483	2.53177	0.07495
H	-1.37944	-1.97577	-1.10400
C	-1.62459	-2.24367	0.99671
H	-1.89310	-1.66498	1.87644
H	-2.34448	-3.05140	0.88395
H	-0.64194	-2.67729	1.16113

IM22

0	2		
C	0.78967	-0.42712	-0.17204
C	1.41551	0.80249	0.02243
C	2.77793	0.90864	0.23464
C	3.54164	-0.24444	0.25039
C	2.94503	-1.47496	0.03589
C	1.57951	-1.56525	-0.18648
C	-0.68081	-0.28947	-0.49261
C	-1.05325	1.12224	-0.14820
H	3.23591	1.87366	0.38648
H	4.60475	-0.17999	0.42305
H	3.54405	-2.37222	0.03295
H	1.14762	-2.53103	-0.38496
S	0.32757	2.17193	-0.06991
C	-2.33912	1.50829	0.07544

C	-1.70578	-1.27176	0.00735
C	-3.33438	0.53303	0.18478
C	-2.97195	-0.82935	0.22382
H	-3.73247	-1.55616	0.47257
H	-4.35838	0.82252	0.35555
H	-2.57759	2.54844	0.23874
C	-1.37895	-2.71946	0.10054
H	-0.64048	-2.91657	0.87656
H	-2.27111	-3.29171	0.33813
H	-0.72964	-0.35934	-1.59926
H	-0.97406	-3.10587	-0.83684

IM23

0	2		
C	0.94915	0.31354	-0.53099
C	1.45059	-0.89479	-0.04770
C	2.70509	-0.97991	0.53095
C	3.45419	0.18344	0.62023
C	2.96921	1.39304	0.14585
C	1.70624	1.45866	-0.43261
C	-0.43801	0.08882	-1.06619
C	-0.55203	-1.40172	-0.99196
H	3.09093	-1.91722	0.90177
H	4.43607	0.14248	1.06670
H	3.57383	2.28263	0.22439
H	1.32708	2.40036	-0.80325
H	-0.53085	0.45116	-2.09302
H	-1.49041	-1.93001	-0.96355
N	0.51285	-1.87777	-0.24946
H	0.69185	-2.84988	-0.10176
C	-1.59107	0.37810	1.18804
C	-2.83240	-0.04163	1.45951
C	-3.65439	0.09351	0.26240
C	-2.90635	0.59496	-0.72766
H	-3.18366	-0.42306	2.40456
H	-4.69832	-0.16889	0.20148
H	-3.22603	0.80674	-1.73487
H	-0.74728	0.39099	1.85795
C	-1.51846	0.82485	-0.23329
H	-1.28380	1.89392	-0.27371

IM24

0	2		
C	-1.52941	0.75954	0.10608
C	-1.49031	-0.66147	0.12517
C	-2.63630	-1.41644	0.00121
C	-3.84613	-0.74859	-0.15757
C	-3.90643	0.64458	-0.19830
C	-2.76377	1.40534	-0.07083

C	-0.23239	1.21932	0.29896
C	0.68630	0.05945	0.47948
H	-2.59882	-2.49492	0.01716
H	-4.75521	-1.32134	-0.25626
H	-4.86124	1.13006	-0.32761
H	-2.81172	2.48320	-0.09392
H	0.09033	2.24446	0.34490
H	1.09111	0.03177	1.49914
N	-0.18937	-1.07330	0.24637
H	0.02129	-1.95317	0.67473
C	2.75626	-1.15535	-0.30306
C	3.99808	-0.77460	0.01883
C	4.04991	0.68120	0.07379
C	2.83967	1.17364	-0.21605
H	4.83675	-1.42579	0.20461
H	4.93179	1.25499	0.30806
H	2.56277	2.21383	-0.25670
H	2.40468	-2.16520	-0.43484
C	1.89388	0.05005	-0.47607
H	1.49767	0.09508	-1.49382

IM25

0	2		
C	-0.79603	-0.47273	-0.47664
C	-1.04553	0.86322	-0.15607
C	-2.29865	1.25035	0.29354
C	-3.28291	0.28031	0.41863
C	-3.03899	-1.04366	0.11070
C	-1.78279	-1.41997	-0.35028
H	-2.50820	2.27688	0.55143
H	-4.26232	0.57049	0.76939
H	-3.82261	-1.77714	0.21640
H	-1.58660	-2.45187	-0.60046
C	1.14450	0.86730	-0.92375
H	1.41263	1.21876	-1.91890
C	0.61916	-0.59546	-0.95432
H	0.66932	-1.03222	-1.95227
C	2.41526	0.74550	-0.01677
H	3.12214	1.56195	-0.11807
C	1.59555	-1.34473	-0.01753
H	1.57122	-2.42416	-0.11411
C	2.87976	-0.64082	-0.35704
H	3.85397	-1.02029	-0.07958
C	1.41298	-0.77899	1.37161
H	0.90266	-1.27686	2.17957
C	1.89953	0.46226	1.37544
H	1.85694	1.17338	2.18448
N	0.07341	1.64742	-0.34038
H	-0.05411	2.61372	-0.64033

IM26

0	2		
C	-0.82514	-0.46426	-0.39906
C	-1.18150	0.87037	-0.09628
C	-2.47647	1.20699	0.24374
C	-3.42494	0.19291	0.29685
C	-3.08505	-1.13069	0.03631
C	-1.78788	-1.46814	-0.30407
H	-2.74384	2.22498	0.48374
H	-4.44354	0.43851	0.55581
H	-3.84210	-1.89699	0.09579
H	-1.52484	-2.49300	-0.51806
C	1.02983	0.95534	-0.72954
H	1.23196	1.32674	-1.74161
C	0.55185	-0.46815	-0.73049
C	2.37836	0.82577	0.02127
H	3.01586	1.70234	-0.03251
C	1.69887	-1.30701	-0.22272
H	1.70529	-2.35836	-0.48609
C	2.88656	-0.44733	-0.66684
H	3.83043	-0.79307	-0.25458
C	1.67883	-0.97231	1.25871
H	1.26342	-1.61085	2.02146
C	2.07748	0.29071	1.39542
H	2.07099	0.87601	2.29937
N	-0.06539	1.68362	-0.13101
H	-0.19288	2.64689	-0.37679
H	2.96221	-0.35368	-1.74942

IM27

0	2		
C	-0.82469	-0.49852	-0.43416
C	-1.12221	0.84910	-0.22120
C	-2.38185	1.25771	0.18046
C	-3.34720	0.28102	0.37540
C	-3.06953	-1.06010	0.16212
C	-1.80121	-1.45154	-0.25362
H	-2.60975	2.30071	0.33953
H	-4.33521	0.57586	0.69516
H	-3.83946	-1.79954	0.31668
H	-1.58456	-2.49591	-0.42549
C	1.07817	0.83098	-0.85085
C	0.59869	-0.60461	-0.88656
H	0.66255	-1.04538	-1.88446
C	2.38192	0.83313	-0.06494
H	3.04051	1.68146	-0.21802
C	1.65863	-1.29162	0.02360
H	1.66956	-2.37459	-0.04402

C	2.90835	-0.54820	-0.45818
H	3.80012	-0.82633	0.09687
C	1.50987	-0.70658	1.40254
H	1.02547	-1.19425	2.23228
C	1.94604	0.55079	1.35738
H	1.87475	1.28812	2.14121
N	-0.00832	1.61522	-0.49435
H	0.06235	2.58256	-0.25290
H	3.08204	-0.64865	-1.52832

IM28

0	2		
C	0.97896	0.55869	-0.05697
C	1.27115	-0.80266	-0.20781
C	2.57691	-1.25911	-0.12505
C	3.57631	-0.33422	0.12923
C	3.29359	1.01658	0.30020
C	1.98856	1.46722	0.20900
H	2.81033	-2.30621	-0.24304
H	4.59866	-0.67394	0.19838
H	4.09426	1.71147	0.49781
H	1.76107	2.51590	0.32877
C	-0.97435	-0.62250	-0.68333
C	-0.44882	0.70791	-0.21522
C	-2.31405	-0.96762	-0.05959
H	-2.74725	-1.81078	-0.59648
C	-1.29425	1.72905	-0.03186
H	-0.94606	2.70992	0.25434
C	-2.24998	-1.27597	1.39193
C	-2.71413	1.48585	-0.18135
H	-3.38576	2.33138	-0.20739
C	-3.21159	0.24187	-0.21705
H	-4.27868	0.07987	-0.24071
N	0.11546	-1.53307	-0.38707
H	0.17175	-2.38685	-0.90910
H	-1.61034	-0.68786	2.03134
H	-3.01367	-1.88242	1.85140
H	-1.13243	-0.53954	-1.76868

IM29

0	2		
C	0.85585	0.34558	-0.35939
C	1.29544	-0.91764	0.03128
C	2.62321	-1.17098	0.30957
C	3.51684	-0.11361	0.20038
C	3.09519	1.14887	-0.18144
C	1.75373	1.38051	-0.46980
H	2.96006	-2.15244	0.60619
H	4.55975	-0.28306	0.42060

H	3.80856	1.95449	-0.25401
H	1.42192	2.36416	-0.76764
C	-0.95783	-1.11272	-0.13871
C	-0.61321	0.26220	-0.64192
H	-0.77835	0.24556	-1.72681
C	-2.23133	-1.49455	0.02164
H	-2.50345	-2.49321	0.32516
C	-1.55181	1.31602	-0.07483
H	-1.40935	2.23917	-0.63383
C	-1.37494	1.57744	1.37761
C	-2.97009	0.81910	-0.26510
H	-3.75966	1.55021	-0.34758
C	-3.25348	-0.48898	-0.19213
H	-4.28258	-0.81284	-0.24981
N	0.21746	-1.79017	0.07554
H	0.25288	-2.67090	0.54850
H	-1.29206	0.74162	2.05601
H	-1.62530	2.54099	1.79142

IM30

0	2		
C	-2.53490	1.76834	-0.04647
C	-3.12163	0.55062	-0.22122
C	-2.36552	-0.73604	-0.32889
C	-0.91322	-0.46496	-0.21249
C	-0.33859	0.77524	-0.03705
C	-1.14417	1.93171	0.04906
H	-3.16741	2.64120	0.02196
H	-4.19762	0.47982	-0.28837
H	-0.70757	2.90734	0.18627
C	1.29055	-0.81649	-0.12944
C	2.55697	-1.37999	-0.12365
C	3.63059	-0.52832	0.03386
C	3.44664	0.84861	0.18237
C	2.18262	1.40067	0.17631
C	1.08195	0.56430	0.01842
H	2.70164	-2.44354	-0.23839
H	4.63071	-0.93325	0.04254
H	4.30810	1.48682	0.30344
H	2.04890	2.46576	0.29146
N	0.06268	-1.41436	-0.26695
H	-0.09010	-2.39520	-0.38425
C	-2.85024	-1.76106	0.70170
H	-3.91793	-1.93304	0.58815
H	-2.34625	-2.71854	0.58013
H	-2.66536	-1.40090	1.71008
H	-2.56216	-1.17179	-1.31941

IM31

0	2		
C	1.08119	0.56257	0.09498
C	1.22100	-0.83389	0.02886
C	2.46670	-1.42188	-0.09825
C	3.57613	-0.59416	-0.16905
C	3.45069	0.79102	-0.12430
C	2.20435	1.37583	0.00142
H	2.57313	-2.49467	-0.15176
H	4.55651	-1.03542	-0.26422
H	4.33237	1.40943	-0.18418
H	2.10450	2.44997	0.04317
C	-0.98853	-0.46395	0.50067
C	-0.31710	0.83973	0.21270
C	-2.37668	-0.56271	-0.03494
C	-1.06260	1.97858	0.03878
H	-0.58069	2.93800	-0.07776
C	-2.96455	-1.91430	-0.20322
C	-2.44279	1.86931	-0.09822
H	-3.04015	2.75539	-0.24274
C	-3.05825	0.59388	-0.21283
H	-4.10076	0.55059	-0.49583
N	-0.01614	-1.45206	0.05637
H	-0.05820	-2.35603	0.49005
H	-2.39258	-2.49210	-0.92926
H	-3.99514	-1.86107	-0.54151
H	-2.94527	-2.47470	0.73477
H	-1.07805	-0.54517	1.60517

IM32

0	2		
C	0.82724	0.33586	-0.12558
C	1.38431	-0.94445	0.09014
C	2.75308	-1.15002	0.16357
C	3.57444	-0.05128	0.01079
C	3.04680	1.22420	-0.21442
C	1.68663	1.42628	-0.28444
H	3.16477	-2.13376	0.33114
H	4.64444	-0.18065	0.06255
H	3.71930	2.05905	-0.33598
H	1.29102	2.41387	-0.46641
C	-0.83787	-1.17300	0.04243
C	-0.58273	0.17498	-0.14578
C	-2.12698	-1.71865	0.04502
H	-2.29859	-2.77290	0.19016
C	-1.67324	1.17276	-0.30790
H	-1.52138	1.71575	-1.24960
C	-1.66153	2.22563	0.80924
C	-3.00888	0.50430	-0.36297
H	-3.86440	1.14404	-0.52603

C	-3.19663	-0.82760	-0.17734
H	-4.20079	-1.22412	-0.20388
N	0.35529	-1.83610	0.19222
H	0.45252	-2.82100	0.32724
H	-1.82112	1.74982	1.77324
H	-0.71034	2.75002	0.84321
H	-2.45030	2.95847	0.65226

IM33

0	2		
C	0.56624	-0.15575	-0.73108
C	1.44908	-0.72829	0.19097
C	2.50077	-0.02174	0.74794
C	2.66332	1.29108	0.34551
C	1.80278	1.87715	-0.57841
C	0.74513	1.16224	-1.11446
C	-0.44687	-1.15526	-1.04985
C	0.03229	-2.34125	-0.44881
H	3.17223	-0.47413	1.46155
H	3.47733	1.87018	0.75385
H	1.95984	2.90268	-0.87371
H	0.06912	1.62117	-1.81789
H	-0.93775	-1.18746	-2.00854
H	-0.39135	-3.32756	-0.46515
N	1.07569	-2.03990	0.37135
H	1.55381	-2.69572	0.95442
C	-2.10480	-0.64976	-0.06364
C	-1.66839	-0.31004	1.27923
C	-1.71691	1.03593	1.41039
C	-2.25399	1.60193	0.19189
C	-2.52613	0.59577	-0.67362
H	-1.40357	1.60492	2.26977
H	-2.40626	2.65400	0.01454
H	-2.93950	0.68950	-1.66401
H	-2.62640	-1.57318	-0.26969
H	-1.31228	-1.01721	2.00915
